# Biological properties and characterization of several variations of a clinical human plasma-based skin substitute model and its manufacturing process

**DOI:** 10.1093/rb/rbae115

**Published:** 2024-09-26

**Authors:** Álvaro Sierra-Sánchez, Jorge Cabañas-Penagos, Sandra Igual-Roger, Luis Martínez-Heredia, Olga Espinosa-Ibáñez, Raquel Sanabria-de la Torre, María I Quiñones-Vico, Ana Ubago-Rodríguez, Antonio Lizana-Moreno, Ana Fernández-González, Jorge Guerrero-Calvo, Natividad Fernández-Porcel, Arena Ramírez-Muñoz, Salvador Arias-Santiago

**Affiliations:** Andalusian Network of Design and Translation of Advanced Therapies, Unidad de Producción Celular e Ingeniería Tisular, Virgen de las Nieves University Hospital, Granada, 18014, Spain; Instituto de Investigación Biosanitaria ibs.GRANADA, Granada, 18012, Spain; Department of Dermatology, Virgen de las Nieves University Hospital, Granada, 18012, Spain; Andalusian Network of Design and Translation of Advanced Therapies, Unidad de Producción Celular e Ingeniería Tisular, Virgen de las Nieves University Hospital, Granada, 18014, Spain; Andalusian Network of Design and Translation of Advanced Therapies, Unidad de Producción Celular e Ingeniería Tisular, Virgen de las Nieves University Hospital, Granada, 18014, Spain; Andalusian Network of Design and Translation of Advanced Therapies, Unidad de Producción Celular e Ingeniería Tisular, Virgen de las Nieves University Hospital, Granada, 18014, Spain; Instituto de Investigación Biosanitaria ibs.GRANADA, Granada, 18012, Spain; Andalusian Network of Design and Translation of Advanced Therapies, Unidad de Producción Celular e Ingeniería Tisular, Virgen de las Nieves University Hospital, Granada, 18014, Spain; Instituto de Investigación Biosanitaria ibs.GRANADA, Granada, 18012, Spain; Andalusian Network of Design and Translation of Advanced Therapies, Unidad de Producción Celular e Ingeniería Tisular, Virgen de las Nieves University Hospital, Granada, 18014, Spain; Instituto de Investigación Biosanitaria ibs.GRANADA, Granada, 18012, Spain; Department of Biochemistry and Molecular Biology III and Immunology, University of Granada, Granada, 18071, Spain; Andalusian Network of Design and Translation of Advanced Therapies, Unidad de Producción Celular e Ingeniería Tisular, Virgen de las Nieves University Hospital, Granada, 18014, Spain; Instituto de Investigación Biosanitaria ibs.GRANADA, Granada, 18012, Spain; Department of Dermatology, University of Granada, Granada, 18016, Spain; Andalusian Network of Design and Translation of Advanced Therapies, Unidad de Producción Celular e Ingeniería Tisular, Virgen de las Nieves University Hospital, Granada, 18014, Spain; Instituto de Investigación Biosanitaria ibs.GRANADA, Granada, 18012, Spain; Andalusian Network of Design and Translation of Advanced Therapies, Unidad de Producción Celular e Ingeniería Tisular, Virgen de las Nieves University Hospital, Granada, 18014, Spain; Instituto de Investigación Biosanitaria ibs.GRANADA, Granada, 18012, Spain; Andalusian Network of Design and Translation of Advanced Therapies, Unidad de Producción Celular e Ingeniería Tisular, Virgen de las Nieves University Hospital, Granada, 18014, Spain; Instituto de Investigación Biosanitaria ibs.GRANADA, Granada, 18012, Spain; Andalusian Network of Design and Translation of Advanced Therapies, Unidad de Producción Celular e Ingeniería Tisular, Virgen de las Nieves University Hospital, Granada, 18014, Spain; Instituto de Investigación Biosanitaria ibs.GRANADA, Granada, 18012, Spain; Andalusian Network of Design and Translation of Advanced Therapies, Unidad de Producción Celular e Ingeniería Tisular, Virgen de las Nieves University Hospital, Granada, 18014, Spain; Instituto de Investigación Biosanitaria ibs.GRANADA, Granada, 18012, Spain; Andalusian Network of Design and Translation of Advanced Therapies, Unidad de Producción Celular e Ingeniería Tisular, Virgen de las Nieves University Hospital, Granada, 18014, Spain; Instituto de Investigación Biosanitaria ibs.GRANADA, Granada, 18012, Spain; Andalusian Network of Design and Translation of Advanced Therapies, Unidad de Producción Celular e Ingeniería Tisular, Virgen de las Nieves University Hospital, Granada, 18014, Spain; Instituto de Investigación Biosanitaria ibs.GRANADA, Granada, 18012, Spain; Department of Dermatology, Virgen de las Nieves University Hospital, Granada, 18012, Spain; Department of Dermatology, University of Granada, Granada, 18016, Spain

**Keywords:** biomaterial, dermal matrix, fibrin, human plasma, tissue-engineered skin substitute, scaffold

## Abstract

Human plasma is a natural biomaterial that due to their protein composition is widely used for the development of clinical products, especially in the field of dermatology. In this context, this biomaterial has been used as a scaffold alone or combined with others for the development of cellular human plasma-based skin substitutes (HPSSs). Herein, the biological properties (cell viability, cell metabolic activity, protein secretion profile and histology) of several variations of a clinical HPSS model, regarding the biomaterial composition (alone or combined with six secondary biomaterials – serine, fibronectin, collagen, two types of laminins and hyaluronic acid), the cellular structure (trilayer, bilayer, monolayer and control without cells) and their skin tissue of origin (abdominal or foreskin cells) and the manufacturing process [effect of partial dehydration process in cell viability and comparison between submerged (SUB) and air/liquid interface (ALI) methodologies] have been evaluated and compared. Results reveal that the use of human plasma as a main biomaterial determines the *in vitro* properties, rather than the secondary biomaterials added. Moreover, the characteristics are similar regardless of the skin cells used (from abdomen or foreskin). However, the manufacture of more complex cellular substitutes (trilayer and bilayer) has been demonstrated to be better in terms of cell viability, metabolic activity and wound healing protein secretion (bFGF, EGF, VEGF-A, CCL5) than monolayer HPSSs, especially when ALI culture methodology is applied. Moreover, the application of the dehydration, although required to achieve an appropriate clinical structure, reduce cell viability in all cases. These data indicate that this HPSS model is robust and reliable and that the several subtypes here analysed could be promising clinical approaches depending on the target dermatological disease.

## Graphical abstract

**Graphical Abstract:**
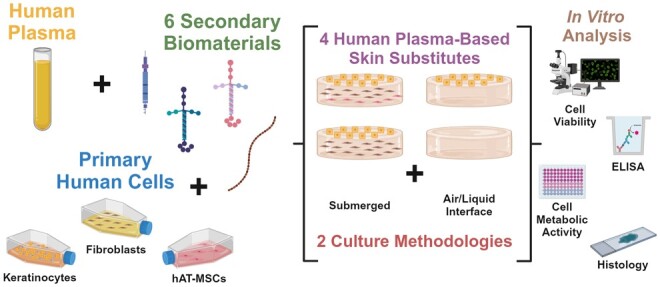
Created in BioRender.com

## Introduction

The human plasma is a light-yellow liquid that carries the blood components throughout the body. Moreover, it is the acellular component of the blood that contains water (90%), proteins, carbohydrates, lipids, salts, enzymes, nutrients or waste products from the blood cells, among others. Therefore, it is constituted of the most complex human-derived proteome and for this reason, it is used for the preparation of many therapeutic products [1].

The usefulness of human plasma as a biomaterial for tissue-engineered skin substitutes (TESSs) lies in the fact that it is an acellular component derived from human donors, so immune rejection is avoided [2]. Moreover, the presence of the clotting proteins fibrinogen (fibrin) and thrombin, together with the *in vitro* application of several procedures such as crosslinking *via* enzymes or UV radiation [3, 4] or the addition of different Ca^2+^ concentrations [5, 6], triggers a gelation process that allows to generate a provisional matrix or hydrogel that closely mimics extracellular matrix (ECM) [7].

In this context, several research groups have explored the use of the human plasma for the development of different TESSs [2, 8–18], referred as human plasma-based skin substitutes (HPSSs). Our group has experience in manufacturing clinical human bilayer tissue-engineered skin substitutes (hbTESSs) based on the use of human plasma as main biomaterial and agarose as secondary biomaterial, combined with human primary keratinocytes and fibroblasts for the treatment of severely burned patients [19, 20]. However, the use of agarose has certain drawbacks such as the necessity to be heated to 39°C just before hbTESS manufacture, which supposes a challenge when working under good manufacturing practice (GMP) conditions and moreover, it is not naturally found in skin or human body.

Regarding the biomaterials used and combined with human plasma for the development of preclinical HPSSs, apart from agarose [19, 21–30], the polyethylene glycol [31–33], the collagen [33, 34] and the hyaluronic acid [28, 35] are the most studied. Among then, collagen and hyaluronic acid are natural components of the human body and for this reason, these types of biomaterials, including laminin or fibronectin could be of interest for the clinical development of HPSSs.

Considering the cellular types used for the development of the HPSSs, human keratinocytes and human fibroblasts [8, 13, 14, 16, 17, 22, 24, 25, 27, 28, 34, 36–41] together with human mesenchymal stem cells (hMSCs) [9, 18, 31–33, 42] have been the most investigated allowing the manufacture of trilayer (three cellular layers), bilayer (two cellular layers) and monolayer (one cellular layer) HPSSs, being the bilayer HPSSs the most investigated for clinical purposes [20, 43–45].

Apart from these aspects and regarding the manufacturing process, the application of a partial dehydration compression over different types of hydrogel-based skin substitutes as a final production step has previously demonstrated its capacity to improve their clinical handling [46–48]. However, the effect of this process on cell viability has only been evaluated in cells within collagen constructs [49] but not in human plasma-based hydrogels. In addition, taking in to account the culture process, it was earlier described that for the development of TESSs with a fully differentiated epidermis, the application of the air/liquid interface (ALI) strategy was required [50]. This is necessary to mimic the physiological process where the basal epidermal cells are exposed to the air, triggering their terminal differentiation, against what happens when the keratinocytes are cultured under submerged (SUB) conditions, where keratinocyte monolayers are obtained [51]. However, although the production of a fully differentiated epidermis would be ideal, for clinical purposes it has been demonstrated that the engraftment of cohesive cultured keratinocyte sheets was enough to heal the wounded skin of patients [52]. This means that the manufacturing time could be decreased since the ALI is not applied, which is a significant asset when the human body is highly exposed due to the lack of our first protective barrier.

Hence, regarding the versatility and possibilities of the HPSSs, the aim of this study was to evaluate *in vitro* the biological properties (cell viability, cell metabolic activity, protein secretion profile and histology) of several variations of our bilayer clinical model and its manufacturing process regarding the secondary biomaterials used and the cellular composition. To that purpose we studied the human plasma as single biomaterial and combined with six secondary biomaterials including serine, fibronectin, collagen, two types of laminins and hyaluronic acid. Moreover, for each of these conditions, trilayer, bilayer and monolayer HPSSs composed of primary human keratinocytes, fibroblasts (both from abdominal skin or foreskin biopsies) and hMSCs, were manufactured by applying two different culture methodologies, submerged and air/liquid interface which were compared. To conclude, the effect on cell viability of the partial dehydration process as a last production stage of these HPSSs was evaluated.

## Material and methods

### Skin cell isolation and culture

Human keratinocytes and fibroblasts were extracted from 11 human skin biopsies (abdomen [*n* = 3] and foreskin [*n* = 8]) from healthy patients (no exclusion was applied regarding factors such as age, body mass index, smoking or alcohol history). Adipose tissue and/or blood traces were removed before starting the one-step digestion protocol applied for skin cell isolation.

Firstly, human skin biopsies were washed for 30 min, submerged in a solution constituted of Dulbecco’s Phosphate Buffered Saline (DPBS; Sigma-Aldrich, St Louis, USA), 160 μg/ml gentamicin (Laboratorios Normon, Madrid, Spain), 100 μg/ml cefotaxime (Medochemie, Limassol, Cyprus), 100 μg/ml vancomycin (Reig Jofre, Barcelona, Spain) and 1.25 μg/ml amphotericin b (Sigma-Aldrich). Then, the samples were transferred to a 60 × 15 mm Petri dish (Fisherbrand^®^, Waltham, MA, USA) with the epidermis facing down. Most of the dermis was mechanically detached using scissors and a scalpel and kept for fibroblast isolation.

#### Epithelial cell isolation

Epidermis was mechanically fragmented into small pieces using scissors and incubated for 15 min in a stirring 5 ml of TrypLE Select Enzyme 10X (Gibco™, Waltham, MA, USA) at 37°C (eight cycles). After each cycle, digested solution containing the cells and pieces of tissue were filtered using a Cell Strainer of 100 µm (Fisherbrand^®^). The filtered cell suspension was neutralized using 10 ml of keratinocyte medium [Dulbecco’s Modified Eagle’s Medium (DMEM) (Sigma-Aldrich): Ham’s F12 (Sigma-Aldrich), ratio 2:1, supplemented with 584 μg/ml L-glutamine (Sigma-Aldrich), 96 μg/ml gentamicin (Laboratorios Normon), 24 μg/ml adenine hydrochloride hydrate (Sigma-Aldrich), 5.25 μg/ml human insulin solution (Sigma-Aldrich), 1.25 μg/ml amphotericin b (Sigma-Aldrich), 400 ng/ml hydrocortisone (Sigma-Aldrich), 25 ng/ml human epidermal growth factor (Sigma-Aldrich), 1.4 ng/ml triiodo-L-thyronine sodium salt (Sigma-Aldrich) and 10% fetal bovine serum (Gibco™)] and kept, while remaining tissue was digested again. After the 8th cycle, the cell suspension was centrifuged (10 min/24°C/1500 rpm). Cells were counted and seeded at 1.3 × 10^5^ cells/cm^2^ on a *feeder* layer of irradiated mouse embryonic fibroblasts (4 × 10^4^ cells/cm^2^) and cultured in keratinocyte medium. This primary culture was named passage 0 (P0).

#### Dermal cell isolation

Dermis was fragmented using scissors and digested using a stirring solution of 2 mg/ml of Type I collagenase (Gibco™) in DMEM (Sigma-Aldrich) for 20 h at 37°C. The digested tissue was filtered using a Steriflip-GP Sterile Centrifuge Tube Top Filter Unit of 0.22 µm (Merck Millipore, Burlington, MA, USA), neutralized using fibroblast medium [DMEM (Sigma-Aldrich) supplemented with 584 μg/ml L-glutamine (Sigma-Aldrich), 96 μg/ml gentamicin (Laboratorios Normon) and 10% fetal bovine serum (Gibco™)] and centrifuged (10 min/24°C/1500 rpm). Finally, dermal cells were counted and seeded at 1.15 × 10^5^ cells/cm^2^ in fibroblast medium. This primary culture was named passage 0 (P0).

#### Culture maintenance and passages

The culture medium of keratinocytes and fibroblasts was changed every two or three days until cells reached 90–95% confluence; then cells were trypsinized and cultured again until reached the enough number of cells for HPSS manufacturing. From P1 cell seeding density for keratinocytes was 7.5 × 10^3^ cells/cm^2^ on a *feeder* layer of irradiated mouse embryonic fibroblasts (10^4^ cells/cm^2^) and for fibroblasts was 3.5 × 10^3^ cells/cm^2^.

### Human adipose tissue mesenchymal stem cell isolation and culture

The human mesenchymal stem cells were extracted, as previously described [53], from one donor subcutaneous adipose tissue sample (*n* = 1). Before starting the isolation protocol, blood traces were removed. Briefly, the subcutaneous fat was washed for 30 min with a solution constituted of DPBS; (Sigma-Aldrich), 160 μg/ml gentamicin (Laboratorios Normon), 100 μg/ml cefotaxime (Medochemie, Limassol, Cyprus) and 100 μg/ml vancomycin (Reig Jofre). Then, the sample was cut in small pieces with scissors to mechanically disaggregate them using a mincer. After that, the grinding tissue was enzymatically digested in a stirring solution of 1 mg/ml of Type A collagenase (Hoffmann-La Roche, Basel, Switzerland) at 37°C for 2 h. The result of the digestion was neutralized with MSC medium [DMEM (Sigma-Aldrich) supplemented with 869 μg/ml alanine-glutamine (Sigma-Aldrich), 96 μg/ml gentamicin (Laboratorios Normon), 50 μg/ml vancomycin (Reig Jofre) and 10% fetal bovine serum (Gibco™)] and after several filtration and centrifugation processes, human adipose tissue mesenchymal stem cells (hAT-MSCs) were counted and cultured at 1.5 × 10^4^ cells/cm^2^ in MSC medium. This primary culture was named passage 0 (P0).

The culture medium of hAT-MSCs was changed every three days until cells reached 90–95% confluence; then cells were trypsinized, seeded at 3.5 × 10^3^ cells/cm^2^ and expanded until reached the end P1 or P2. In that moment, hAT-MSCs were cryopreserved in a solution constituted of Fetal Bovine Serum (Gibco™) and 10% dimethyl sulfoxide (Sigma-Aldrich). Finally, when required, hAT-MSCs were thawed and expanded until reached the enough number of cells for HPSS manufacturing.

### HPSS manufacture

The 11 human skin cell populations (keratinocytes and fibroblasts) were used for manufacturing all type of cellular HPSSs that will be described in subsequent sections. The future application of hAT-MSCs in this clinical TESS model is supposed to be allogeneic, for this reason the same cell population (previously aliquoted and frozen) was used for manufacturing the HPSSs in those cases where a hypodermal layer was included.

#### Dermal matrix composition/tested biomaterials

The HPSS model is based on the use of human plasma/fibrin (Fib) as main biomaterial to produce a hydrogel that will support cell growth and differentiation. Therefore, frozen human blood plasma from healthy donors was collected from the Centro Regional de Transfusión Sanguínea of Granada. Human plasma from different donors were mixed to generate a pool which was aliquoted, frozen at −20°C and used for all experiments to avoid inter-variability between samples.

Apart from the human plasma as a single biomaterial, this was independently combined with six secondary biomaterials to determine their biological properties: serine (S), fibronectin (Fn), collagen (Col), two types of laminins (Lam-1/Lam-2) (all from Biogelx™, Lanarkshire, UK) and hyaluronic acid (HA) (Fidia Farmaceutici S.p.A., Abano Terme, Italy) (Supplementary Table 1). The final concentration of each secondary biomaterial into the hydrogel was stablished after preliminary studies where clinical handling was evaluated (data not shown).

#### Cell layer composition

Regarding cellular structure, four types of HPSSs were produced for each biomaterial combination and human skin cell population: trilayer HPSSs; constituted of a hypodermal layer of 6 × 10^4^ hAT-MSCs/ml, a dermal layer of 6 × 10^4^ human fibroblasts/ml and an epidermal layer constituted of 1.77 × 10^5^ keratinocytes/cm^2^, bilayer HPSSs; comprised of a dermal layer of 6 × 10^4^ human fibroblasts/ml and an epidermal layer constituted of 1.77 × 10^5^ keratinocytes/cm^2^, monolayer HPSSs; which resembled an epidermal layer of 1.77 × 10^5^ keratinocytes/cm^2^ and control HPSSs; as wound dressings without cells.

All TESSs were manufactured in six insert well plates with 0.4 µm pore polyester membrane (Corning^®^ Transwell^®^, Sigma-Aldrich). The final volume was 5 ml/hydrogel and different steps were carried out depending on the cell composition (Supplementary Tables 2 and 3).

Briefly, all reagents were previously warmed at 37°C and then two solutions were prepared in independent tubes (Solutions 1 and 2). For Solution 2, secondary biomaterial and tranexamic acid (MEDA Pharma, Bad Homburg, Germany) were the last reagents to be added to the human plasma and cell suspension. Then, Solution 2 was mixed with Solution 1 [CaCl_2_ (BBraun, Melsungen, Germany) and water (Fresenius Kabi, Bad Homburg, Germany)] and after shaking three times, the mixed solution was carefully pipetted into the six insert well plates. After 2–3 h at 37°C, hydrogel was formed and fibroblast medium was added and incubate overnight (ON) at 37°C (for trilayer HPSSs, this process took place once dermal layer was manufactured).

The following day, keratinocytes were added on top of the corresponding hydrogels in 500 µl of keratinocyte medium. They were allowed to adhere to the surface for 1–2 h and then fibroblast medium was replaced by keratinocyte medium to fully cover the HPSSs.

#### HPSS culture and partial dehydration process

Two culture methodologies were applied for the development of the HPSSs: submerged or air/liquid interface culture. At the end of both strategies, HPSSs were subjected to a partial dehydration process [46] using a sandwich system where absorbent papers and meshes of different pore sizes were used together with a glass disk to apply a pressure of 460 Pa for 2 min that improved their mechanical properties and handling [46].

##### Submerged culture methodology

Seven batches were produced and cultured applying the submerged approach (SUB): three human skin cell populations from abdominal skin (*N* = 3; ABDO) and four from foreskin (*N* = 4; FORE) were used for manufacturing trilayer, bilayer, monolayer and control HPSSs as previously described. Keratinocyte medium was changed every two days for 7–10 days from keratinocyte’s addition and the HPSSs were fully covered to promote cell proliferation and growth.

##### Air/liquid interface culture methodology

Five batches, all of them from different human skin cell populations from foreskin (*N* = 5), were manufactured using the air/liquid interface approach (ALI). Trilayer, bilayer, monolayer and control HPSSs for each skin cell population and secondary biomaterial were also produced but after culturing them for 7–10 days in submerged conditions, then air/liquid interface strategy was applied for 10–13 more days, changing the culture medium every two days. This is based on exposing the epidermal layer to the air while the stromal layers are fully covered with keratinocyte medium exempt of human epidermal growth factor. The purpose was to induce the terminal differentiation of the epithelium but promoting the proliferation and growth of the rest of the layers.

### Biological characterization of the HPSSs

#### Cell viability

Cell viability after manufacturing process of HPSSs was evaluated using LIVE/DEAD™ Viability/Cytotoxicity Kit (Invitrogen™, Waltham, MA, USA); a two-color assay to determine viability of cells based on their esterase activity and plasma membrane integrity. A sample of 8 mm of diameter was taken from each HPPS manufactured, washed three times with DPBS (Sigma-Aldrich) and incubated for 45 min at room temperature in a solution constituted of DPBS and two fluorophores: calcein-AM (1:2000) which emits green fluorescence (517 nm) due to the intracellular esterase activity of viable cells and ethidium homodimer-1 (1:500) that emits red fluorescence (617 nm) when binds to DNA due to the loss of plasma membrane integrity of dead cells.

Then, after washing with DPBS (three times), at least three digital images of each HPSS were taken using a Leica DM2000 microscope coupled with a Leica Mercury Burner 100 W Hg Lamp and a Leica DMC2900 camera (Leica, Wetzlar, Germany). Images were processed using Leica Application Suite (LAS) 4.12 software (Leica).

These digital images were used to count live and dead cells and determine the mean cell viability of each HPSS. In the case of submerged HPSSs, cell viability was determined before (HYD) and after dehydration (DEHYD) process to evaluate the effect of this process (Table 1).

**Table 1.** Conditions analysed and compared for cell viability of the human plasma-based skin substitutes (HPSSs)

Conditions compared	Culture methodology	Regardless of	Total number of replicas considered for each condition compared (*n*)	Number of human skin cell population used (*N*)
Secondary biomaterial used for each type of cellular HPSS *Fib/S vs. Fib/Fn vs. Fib/Col vs. Fib/Lam-1 vs. Fib/Lam-2 vs. Fib/HA vs. Fib*	SUB (hydrated) SUB (dehydrated) ALI (dehydrated)	Skin cell tissue source	7-SUB/HYD 7-SUB/DEHYD 5-ALI/DEHYD	7-SUB/HYD 7-SUB/DEHYD 5-ALI/DEHYD
Partial dehydration process for each type of cellular HPSS *Hydrated (HYD) vs. Dehydrated (DEHYD)* *Trilayer vs. Bilayer vs. Monolayer*	SUB	Secondary biomaterial and skin cell tissue source	49	7
Skin cell tissue source for each type of cellular HPSS *Abdominal skin (ABDO) vs. Foreskin (FORE)* *Trilayer vs. Bilayer vs. Monolayer*	SUB (hydrated & dehydrated)	Secondary biomaterial	21-ABDO 28-FORE	3-ABDO 4-FORE
Culture methodology for each type of cellular HPSS *Submerged culture (SUB) vs. Air/liquid interface culture (ALI)* *Trilayer vs. Bilayer vs. Monolayer*	SUB (dehydrated) ALI (dehydrated)	Secondary biomaterial and skin cell tissue source	49-SUB 35-ALI	7-SUB 5-ALI

#### Cell metabolic activity

Cell metabolic activity of HPSSs was evaluated using PrestoBlue™ (Invitrogen™); a colorimetric assay which allows to measure resazurin reduction level (absorbance at 570 and 600 nm) by living cells. To that purpose, two samples of 5 mm of diameter was taken from each HPSS and incubated at 37°C in a PrestoBlue™-phosphate buffered saline (1/10) solution for 20 h. Then the supernatant was collected, and absorbance was measured using a Multi-mode Reader Synergy™ HTX (BioTek, Winooski, VT, USA). The percentage of reduction was calculated using the following equation:

$$\%\;\mathrm{Reduction}=\;\frac{\left(O2\times A1\right)-\left(O1\times A2\right)}{\left(R1\times C2\right)-\left(R2\times C1\right)}\;\times \;100$$

-
O1 : molar extinction coefficient (*E*) of oxidized PrestoBlue™ (Blue) at 570 nm ≥ 80 586
-
O2 : *E* of oxidized PrestoBlue™ (Blue) at 600 nm ≥ 117 216
-
R1 : *E* of reduced PrestoBlue™ (Red) at 570 nm ≥ 155 677
-
R2 : *E* of reduced PrestoBlue™ (Red) at 600 nm ≥ 14 652
-
A1 : absorbance of test HPSS at 570 nm
-
A2 : absorbance of test HPSS at 600 nm
-
C1 : absorbance of control HPSS at 570 nm
-
C2 : absorbance of control HPSS at 600 nm

The mean value of the two samples taken from each HPSS was used for statistical analysis. These data allowed to compare several conditions (Table 2).

**Table 2.** Conditions analysed and compared for cell metabolic activity of the human plasma-based skin substitutes (HPSSs)

Conditions compared	Culture methodology	Regardless of	Total number of replicas considered for each condition compared (*n*)	Number of human skin cell population used (*N*)
Secondary biomaterial used for each type of cellular HPSS *Fib/S vs. Fib/Fn vs. Fib/Col vs. Fib/Lam-1 vs. Fib/Lam-2 vs. Fib/HA vs. Fib*	SUB ALI	Skin cell tissue source	7-SUB 5-ALI	7-SUB 5-ALI
Skin cell tissue source for each type of cellular HPSS *Abdominal skin (ABDO) vs. Foreskin (FORE)* *Trilayer vs. Bilayer vs. Monolayer*	SUB	Secondary biomaterial	21-ABDO 28-FORE	3-ABDO 4-FORE
Culture methodology for each type of cellular HPSS *Submerged culture (SUB) vs. Air/liquid interface culture (ALI)* *Trilayer vs. Bilayer vs. Monolaye*	SUB ALI	Secondary biomaterial and skin cell tissue source	49-SUB 35-ALI	7-SUB 5-ALI

#### Protein secretion profile analysis

For protein secretion profile analysis, the culture medium surrounding the HPSSs was collected and stored at −80°C at the end of the manufacturing process for further analysis. Three human growth factors (bFGF, EGF and VEGF) and one cytokine (CCL5), involved in wound healing process [54–56], were measured and quantified using commercial kits based on Sandwich-ELISA (Enzyme-Linked Immunosorbent Assay) principle (Elabscience^®^, Houston, TX, USA).

Briefly, for each protein analysed, culture medium was incubated in wells precoated with the specific antibody, then a secondary biotinylated detection antibody specific for the protein of interest and avidin-horseradish peroxidase (HRP) conjugated were added successively to each micro-plate well and incubated. After several washes, the substrate solution was added to each well and enzyme-substrate reaction was terminated by the addition of stop solution. Those wells containing the protein of interested showed a yellow color. The optical density (OD) was measured spectrophotometrically at a wavelength of 450 ± 2 nm using a Multi-mode Reader Synergy™ HTX (BioTek). The concentration was determined by comparing the OD of the samples to the standard curve, using the GainData^®^ software (Arigo, Hsinchu City, Taiwan).

Several comparisons were analysed (Table 3), however in the case where submerged and air/liquid interface culture methodologies were directly compared, the culture medium collected and analysed from submerged samples was the one surrounding the same HPSSs that were subsequently cultured by the air/liquid interface strategy.

**Table 3.** Conditions analysed and compared for each protein analysed by ELISA

Conditions compared	Culture methodology	Regardless of	Total number of replicas considered for each condition compared (*n*)	Number of human skin cell population used (*N*)
Secondary biomaterial used for each type of cellular HPSS *Fib/S vs. Fib/Fn vs. Fib/Col vs. Fib/Lam-1 vs. Fib/Lam-2 vs. Fib/HA vs. Fib*	SUB ALI	Skin cell tissue source	5-SUB 5-ALI	5-SUB 5-ALI
Skin cell tissue source for each type of cellular HPSS *Abdominal skin (ABDO) vs. Foreskin (FORE)* *Trilayer vs. Bilayer vs. Monolayer*	SUB	Secondary biomaterial	21-ABDO 28-FORE	3-ABDO 4-FORE
Culture methodology for each type of cellular HPSS *Submerged culture (SUB) vs. Air/liquid interface culture (ALI)* *Trilayer vs. Bilayer vs. Monolayer*	SUB ALI	Secondary biomaterial and skin cell tissue source	35-SUB 35-ALI	5-SUB 5-ALI

To normalize the results, the corresponding control HPSS values were subtracted to the values of the rest of cellular HPSSs manufactured and then, this value was divided by the initial number of seeded cells for each condition and human skin cell population used. The subtraction of the control HPSS values is important because the own human plasma is a source of several factors and cytokines, some of which are the ones included in this study.

#### Histological analysis

For histological analysis, only dehydrated samples were fixed in formaldehyde 4% (pH = 7) (Casa Álvarez, Madrid, Spain) and embedded in paraffin (Thermo Fisher Scientific, Waltham, MA, USA). Five-micrometer thick sections were stained with hematoxylin/eosin (Casa Álvarez). Digital images were acquired using a Leica DM2000 microscope coupled with Leica DMC2900 (brightfield) camera (Leica). Images were processed using Leica Application Suite (LAS) 4.12 software (Leica).

##### Epidermal thickness analysis

To compare the epidermal development as a result of the submerged and air/liquid interface methodologies, the thickness of the epidermis was measured from a minimum of six regions from each picture to calculate the mean value thickness, using the ImageJ software (NIH, Bethesda, MD, USA).

### Statistical analysis

Microsoft Excel and Adobe Photoshop 2020 software were used for primary analysis of raw data, and GraphPad Prism 8 (San Diego, CA, USA) was used for statistical analysis and graphs. Normal distribution of the data was evaluated by Shapiro–Wilk test. For biomaterial comparison if normal distribution was confirmed, the results were analysed by one-way ANOVA and Tukey’s multiple comparisons tests, however if no normal distribution was corroborated, Friedman and Dunn’s multiple comparisons tests were performed. To compare different conditions of the same biomaterial, multiple *t*-test and Holm–Sidak’s method were used to analyse the results. For the rest of conditions and groups compared, when normal distribution was corroborated, Welch’s *t*-test was performed. However, when no normal distribution was confirmed, Mann–Whitney test was used for analysis of the results.

All values were presented as mean value ± standard deviation (SD). The significance threshold was set at 0.05.

### Ethics

This study was conducted according to the Declaration of Helsinki and was approved by the Provincial Ethics Committees of Granada for human subjects (Project: Tesis-Piel-2020). All tissue donors provided informed written consent.

## Results

### Analysis of secondary biomaterials used for HPSS manufacture

Six secondary biomaterials combined with human plasma/fibrin were used for manufacturing four types of cellular HPSSs (trilayer, bilayer, monolayer and control): Fib/S, Fib/Fn, Fib/Col, Fib/Lam-1, Fib/Lam-2 and Fib/HA. Skin substitutes without a secondary biomaterial were also manufactured (Fib).

#### Cell viability and cell metabolic activity

**Figure 1.**
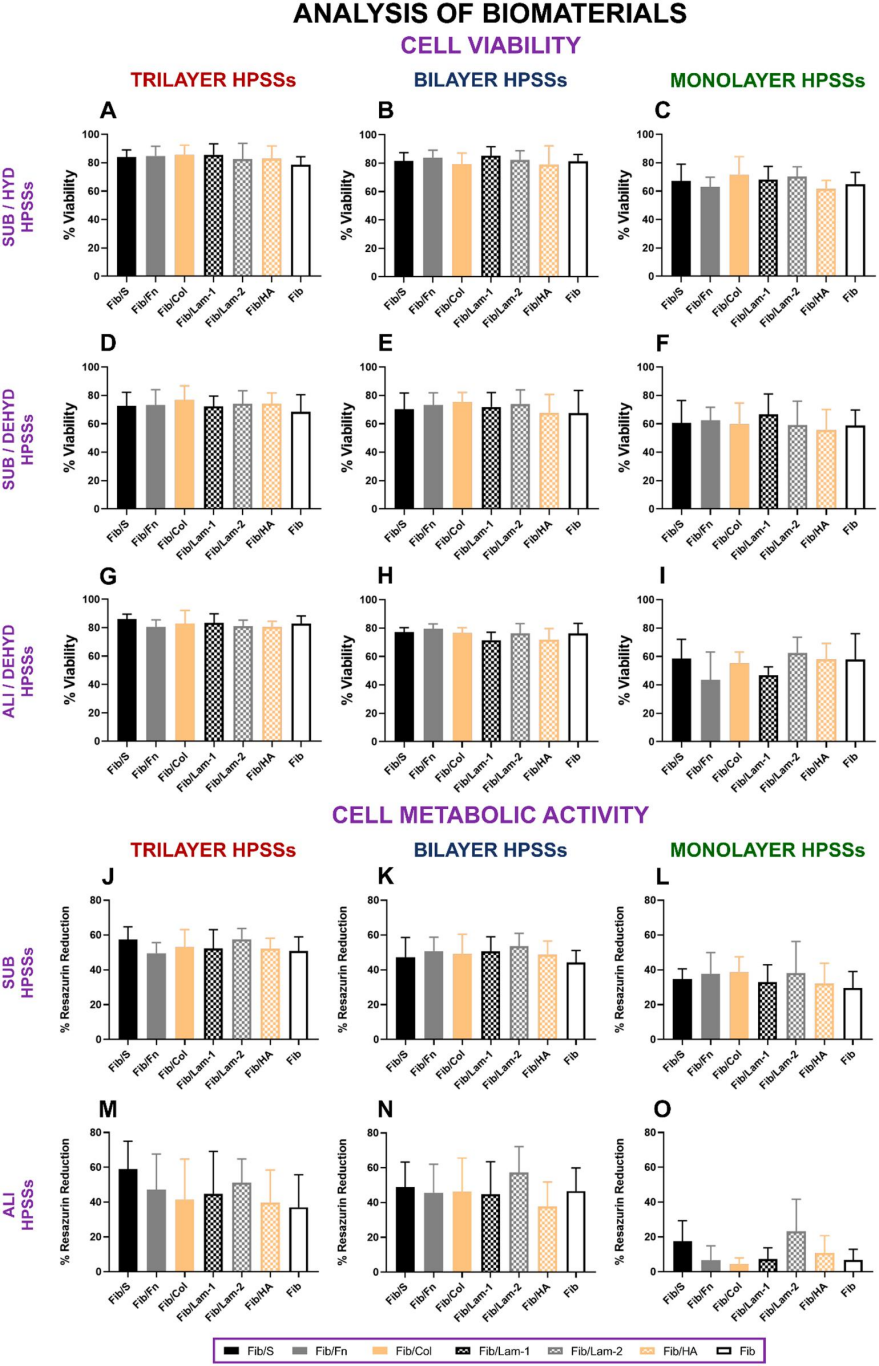
Cell viability and cell metabolic activity analysis of human plasma-based skin substitutes (HPSSs) manufactured with several secondary biomaterials. (**A**, **B** and **C**) Cell viability of trilayer, bilayer and monolayer HPSSs, respectively, cultured under submerged condition and before partial dehydration process; (**D**, **E** and **F**) Cell viability of trilayer, bilayer and monolayer HPSSs, respectively, cultured under submerged condition and after partial dehydration process; (**G**, **H** and **I**) Cell viability of trilayer, bilayer and monolayer HPSSs, respectively, cultured under air/liquid interface condition and after partial dehydration process; (**J**, **K** and **L**) Cell metabolic activity of trilayer, bilayer and monolayer HPSSs, respectively, cultured under submerged condition; (**M**, **N** and **O**) Cell metabolic activity of trilayer, bilayer and monolayer HPSSs, respectively, cultured under air/liquid interface condition. Statistical analysis: one-way ANOVA and Tukey’s multiple comparisons tests (if normal distribution) or Friedman and Dunn’s multiple comparisons tests (if no normal distribution). Data are shown as mean value ± SD; *N* = 7 for submerged conditions (*n* = 7) and *N* = 5 for air/liquid interface conditions (*n* = 5). Fib/S: fibrin/serine; Fib/Fn: fibrin/fibronectin; Fib/Col: fibrin/collagen; Fib/Lam-1: fibrin/laminin-1; Fib/Lam-2: fibrin/laminin-2; Fib/HA: fibrin/hyaluronic acid; Fib: fibrin. HYD: hydrated; DEHYD: dehydrated. SUB: submerged; ALI: air/liquid interface.

Cell viability results revealed that no significant differences were observed between secondary biomaterials used (Figure 1A–I). Despite the several conditions analysed for each of the cellular HPSSs developed, such as hydrated (HYD) (Figure 1A–C) and dehydrated (DEHYD) (Figure 1D–F) conformation or the application of submerged (SUB) (Figure 1D–F) and air/liquid interface (ALI) (Figure 1G–I) culture strategies, the *in vitro* role of the secondary biomaterials used in this HPSS model was not that decisive. This was also reported when cell metabolic activity was measured (Figure 1J–O), demonstrating that the added cells presented a similar behavior regardless of the secondary biomaterial used, even when several culture strategies were applied.

#### Protein secretion profile and histological analysis

**Figure 2.**
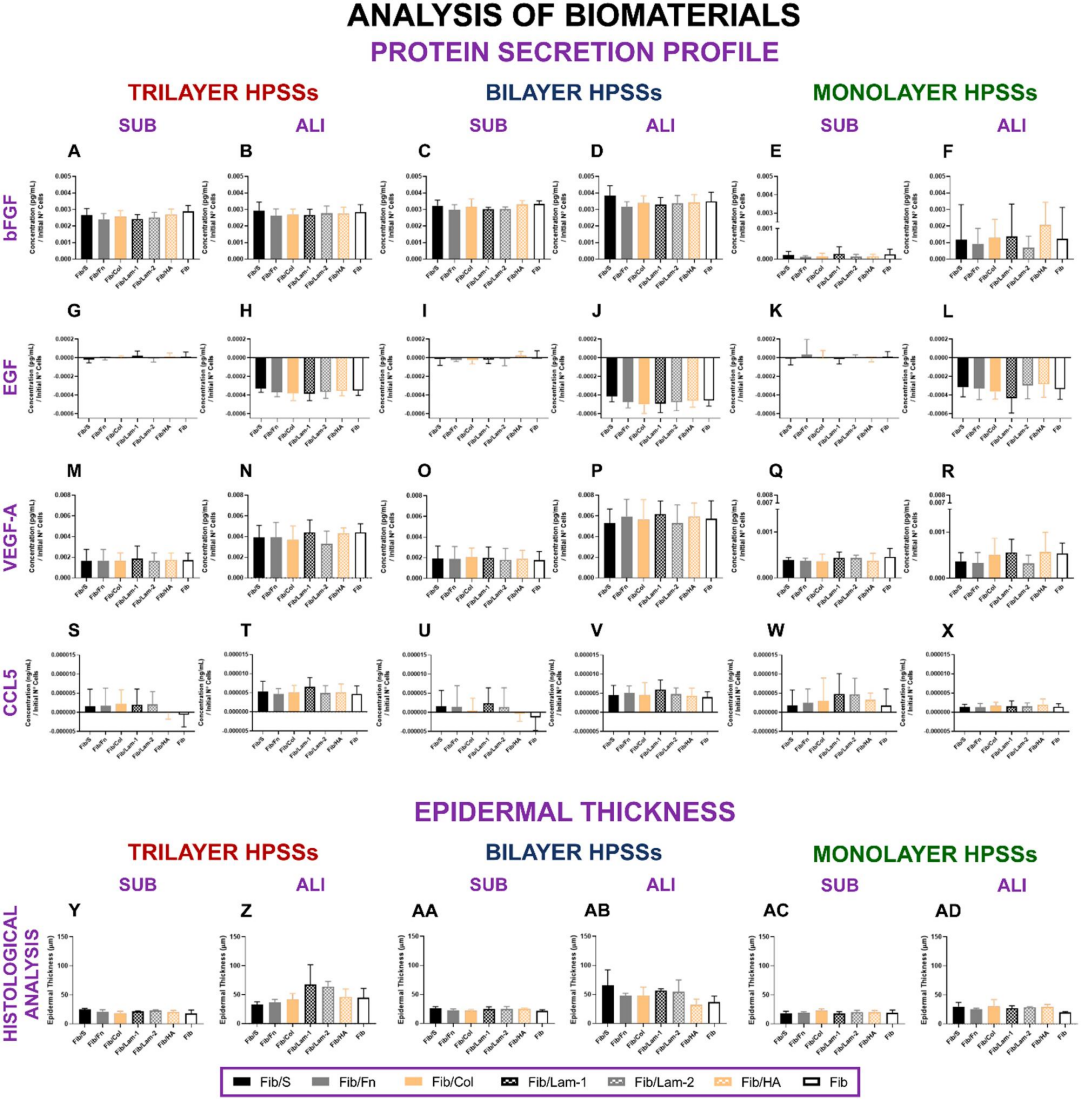
Protein secretion profile of human plasma-based skin substitutes (HPSSs) manufactured with several secondary biomaterials. **bFGF secretion**: (**A** and **B**) Trilayer HPSSs cultured under submerged and air/liquid interface methodologies; (**C** and **D**) Bilayer HPSSs cultured under submerged and air/liquid interface methodologies; (**E** and **F**) Monolayer HPSSs cultured under submerged and air/liquid interface methodologies. **EGF secretion**: (**G** and **H**) Trilayer HPSSs cultured under submerged and air/liquid interface methodologies; (**I** and **J**) Bilayer HPSSs cultured under submerged and air/liquid interface methodologies; (**K** and **L**) Monolayer HPSSs cultured under submerged and air/liquid interface methodologies. **VEGF-A secretion**: (**M** and **N**) Trilayer HPSSs cultured under submerged and air/liquid interface methodologies; (**O** and **P**) Bilayer HPSSs cultured under submerged and air/liquid interface methodologies; (**Q** and **R**) Monolayer HPSSs cultured under submerged and air/liquid interface methodologies. **CCL5 secretion**: (**S** and **T**) Trilayer HPSSs cultured under submerged and air/liquid interface methodologies; (**U** and **V**) Bilayer HPSSs cultured under submerged and air/liquid interface methodologies; (**W** and **X**) Monolayer HPSSs cultured under submerged and air/liquid interface methodologies. **Epidermal thickness**: (**Y** and **Z**) Trilayer HPSSs cultured under submerged and air/liquid interface methodologies; (**AA** and **AB**) Bilayer HPSSs cultured under submerged and air/liquid interface methodologies; (**AC** and **AD**) Monolayer HPSSs cultured under submerged and air/liquid interface methodologies. Statistical analysis: one-way ANOVA and Tukey’s multiple comparisons tests (if normal distribution) or Friedman and Dunn’s multiple comparisons tests (if no normal distribution). Data are shown as mean value ± SD; *N* = 5 for submerged (*n* = 5) and air/liquid interface (*n* = 5) conditions. Fib/S: fibrin/serine; Fib/Fn: fibrin/fibronectin; Fib/Col: fibrin/collagen; Fib/Lam-1: fibrin/laminin-1; Fib/Lam-2: fibrin/laminin-2; Fib/HA: fibrin/hyaluronic acid; Fib: fibrin. SUB: submerged; ALI: air/liquid interface.

Cell viability and metabolic activity results were supported by the protein secretion profile analysis of the growth factors bFGF, EGF and VEGF-A and the cytokine CCL5 (Figure 2A–X). The use of several secondary biomaterials did not demonstrate the release of different amounts of these factors; however, it was confirmed that the cellular complexity and the culture methodology applied played an important role. This was also reported when epidermal thickness was measured for each of the HPSSs manufactured with the several secondary biomaterials (Figure 2Y–AD), elucidating that the culture methodology applied had more influence on the development of the epithelial layer. Representative histological pictures for each secondary biomaterial analysed can be found in Supplementary Figure 1.

Regarding the results of this section, and to reduce the variability of the data, from here, the rest of the conditions were analysed without considering the secondary biomaterial used, focusing on the cellular complexity and comparing the several manufacturing conditions previously indicated.

### Effect of partial dehydration process on cell viability

**Figure 3.**
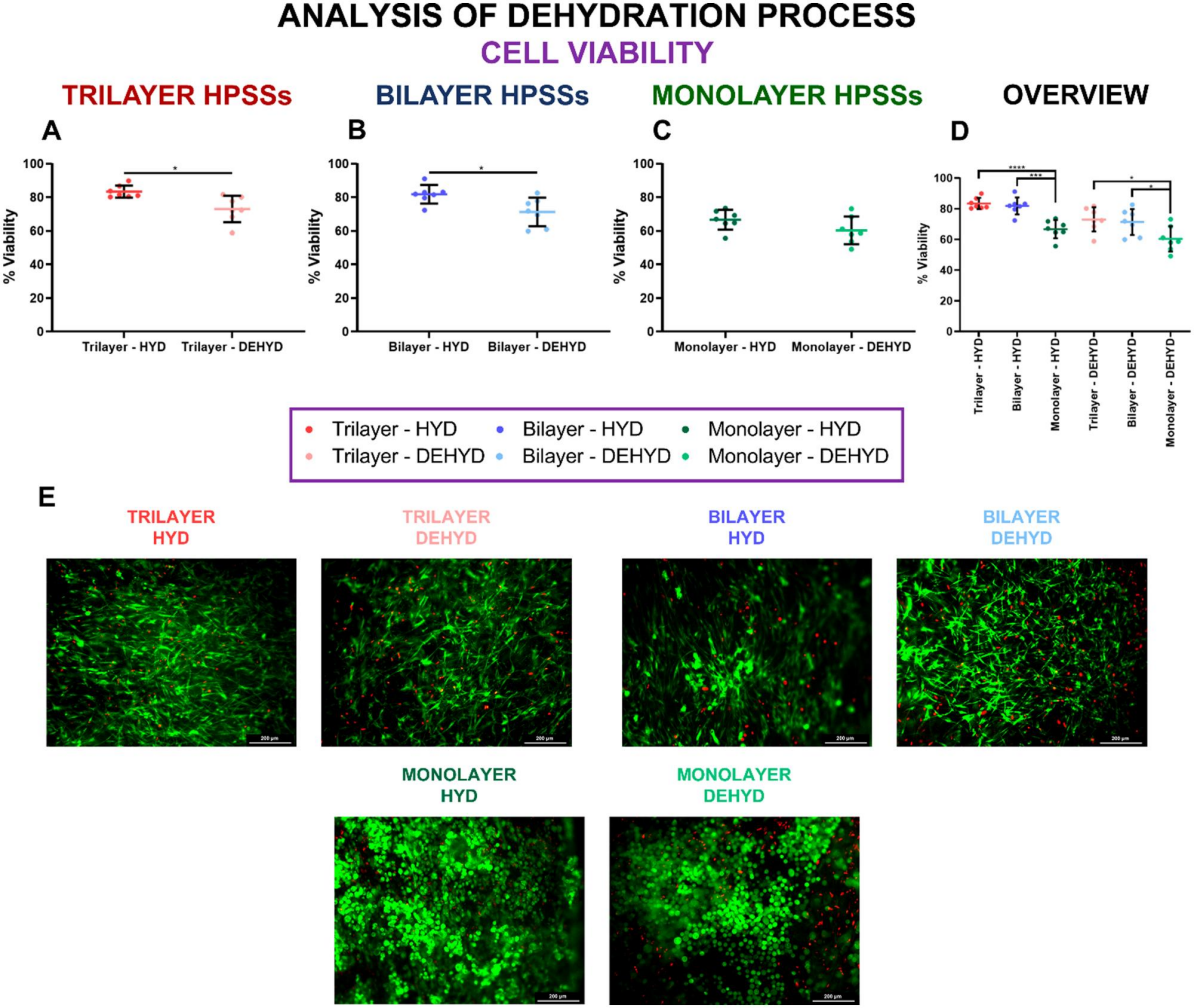
Cell viability analysis of human plasma-based skin substitutes (HPSSs) before and after partial dehydration process. (**A**) Cell viability of trilayer HPSSs; (**B**) Cell viability of bilayer HPSSs; (**C**) Cell viability of monolayer HPSSs; (**D**) Overall analysis of the different cellular HPSSs and their hydration status; (**E**) Representative microscope pictures of LIVE/DEAD™ viability/cytotoxicity assays. Scale bar: 200 µm. Statistical significance: *P values < 0.05, ***P values < 0.001, ****P values < 0.0001, Welch’s *t*-test. Data are shown as mean value ± SD; *N* = 7 for hydrated (*n* = 49) and dehydrated (*n* = 49) conditions. HYD: hydrated; DEHYD: dehydrated.

The effect of partial dehydration process applied after culturing the HPSSs was evaluated on cell viability. To that purpose, HPSSs manufactured with seven different skin cell populations (*N* = 7) and cultured under SUB methodology were studied and compared before (HYD) and after (DEHYD) dehydration process was applied (Figure 3).

The direct comparison of the different cellular HPSSs manufactured, revealed that partial dehydration process reduced cell viability in trilayer, bilayer and monolayer HPSSs (Figure 3A–C), respectively. This decrease was statistically significant in trilayer and bilayer HPSSs (*P* values* < *0.05) (Figure 3A and B), while a slight reduction was reported in monolayer HPSSs (from 66.7 ± 5.9% to 60.3 ± 8.3%) (Figure 3C) but without a significant difference.

Moreover, an overall analysis and comparison of the different cellular HPSSs for each level of hydration revealed that viability was significantly greater for trilayer and bilayer substitutes compared with monolayer HPSSs, in both conditions (Figure 3D). These differences were higher in hydrated status (*P* values* < *0.0001 and *P* values* < *0.001 for trilayer and bilayer groups, respectively) than in dehydrated condition (*P* values* < *0.05 for both trilayer and bilayer HPSSs). Representative microscope pictures of LIVE/DEAD™ Viability/Cytotoxicity assays are included in Figure 3E. In these, a circular or polygonal cell morphology was observed in monolayer HPSSs compared to the more spindle shaped cells presented in trilayer and bilayer substitutes. This was expected in the case of the monolayer group since keratinocytes are known to have a cubic shape, mainly at the basal layer of the epidermis. On the other hand, in the case of trilayer and bilayer substitutes, the representative pictures are focused on the dermal layers (although the epithelial layer viability was also included into the analysis), where fibroblasts and hAT-MSCs are well known to be spindle cells, as was observed.

### Analysis of skin cell tissue sources used for HPSS manufacture

Two human skin cell tissue sources were used for isolation and culture of keratinocytes and fibroblasts required for HPSS manufacture. These substitutes were cultured following the SUB methodology and compared regardless of the biomaterial used. The skin cells were procured from abdominal skin (ABDO; *N* = 3) and foreskin (FORE; *N* = 4) and an individual comparison according to their cell layer composition was provided for each condition evaluated. Moreover, an overview and analysis of the behavior of the different cellular HPSSs, depending on the skin cell tissue source, was also provided.

#### Cell viability and cell metabolic activity

**Figure 4.**
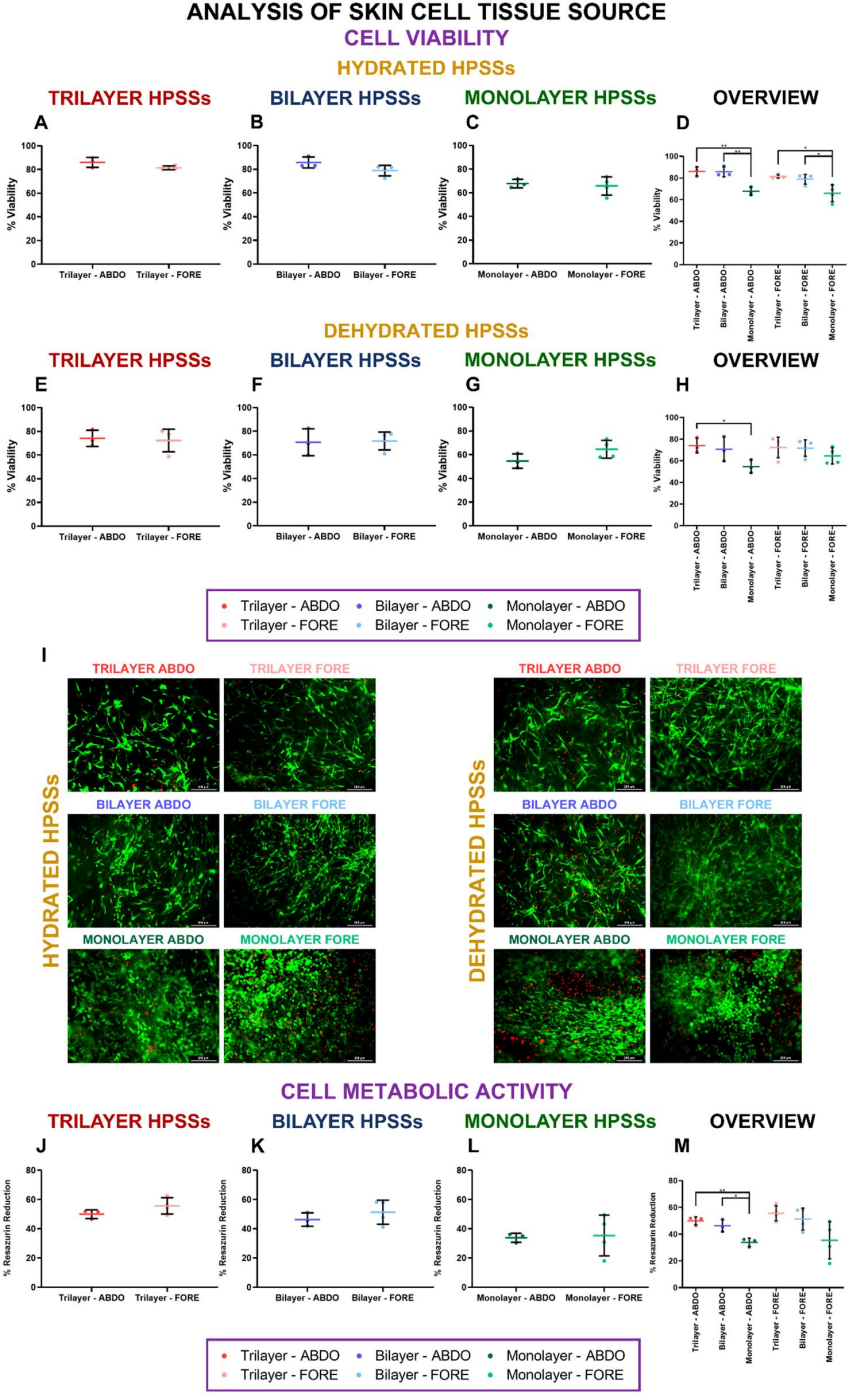
Cell viability and cell metabolic activity analysis of human plasma-based skin substitutes (HPSSs) manufactured with several skin cell tissue sources before and after partial dehydration process. (**A**, **B** and **C**) Cell viability of hydrated (before partial dehydration process) of trilayer, bilayer and monolayer HPSSs, respectively; (**D**) Overall analysis of the different cellular HPSSs and their skin cell tissue source (hydrated); (**E**, **F** and **G**) Cell viability of dehydrated (after partial dehydration process) of trilayer, bilayer and monolayer HPSSs, respectively; (**H**) Overall analysis of the different cellular HPSSs and their skin cell tissue source (dehydrated); (**I**) Representative microscope pictures of LIVE/DEAD™ viability/cytotoxicity assays; (**J**) Cell metabolic activity of trilayer HPSSs; (**K**) Cell metabolic activity of bilayer HPSSs; (**L**) Cell metabolic activity of monolayer HPSSs; (**M**) Overall analysis of the different cellular HPSSs and their skin cell tissue source. Scale bar: 200 µm. Statistical significance: *P values < 0.05, **P values < 0.01, Welch’s *t*-test. Data are shown as mean value ± SD; *N* = 3 for abdominal skin source (*n* = 21) and *N* = 4 for foreskin source (*n* = 28). ABDO: abdominal skin source; FORE: foreskin source.

Cell viability was evaluated before (Figure 4A–D) and after (Figure 4E–H) dehydration process was applied, in addition to the cell metabolic activity at the end of the manufacturing process (Figure 4J–M).

Cell viability analysis of hydrated or dehydrated HPSSs reported similar values when abdominal skin or foreskin cells were used, and no significant differences were observed for any of the cellular groups manufactured; trilayer (Figure 4A and E), bilayer (Figure 4B and F) or monolayer (Figure 4C and G) HPSSs.

An overview of the different cellular groups analysed before dehydration process demonstrated that, for each type of skin cell tissue source, the viability of trilayer and bilayer HPSSs was similar but significantly higher than in monolayer HPSSs (*P* values* < *0.01 for ABDO and *P* values* < *0.05 for FORE) (Figure 4D). In the case of dehydrated HPSSs, viability was also higher for trilayer and bilayer HPSSs, however only a significant difference was reported when trilayer and monolayer HPSSs from ABDO group were analysed (*P* values* < *0.05) (Figure 4H). Representative microscope pictures of LIVE/DEAD™ Viability/Cytotoxicity assays are included in Figure 4I. In these, as in the case of the partial dehydration process analysis, the morphology of the cells was rounded in monolayer HPSSs, due to the single presence of keratinocytes, meanwhile, in the case of trilayer and bilayer HPSSs only pictures from stromal layers were included.

These results correspond with the cell metabolic activity analysis, where no significant differences were observed regarding cell tissue source for trilayer (Figure 4J), bilayer (Figure 4K) or monolayer (Figure 4L) substitutes. Moreover, the overall analysis (Figure 4M) reported that also trilayer and bilayer substitutes reduced resazurin in a higher percentage regardless of abdominal or foreskin cells were used. These results were consistent with the initial number of cells seeded on each type of hTESS. However, only when abdominal skin cells were used, significant differences were reported between trilayer and bilayer groups compared with monolayer HPSSs (*P* values* < *0.01 and *P* values* < *0.05, respectively) (Figure 4M).

#### Protein secretion profile analysis and histological appearance

**Figure 5.**
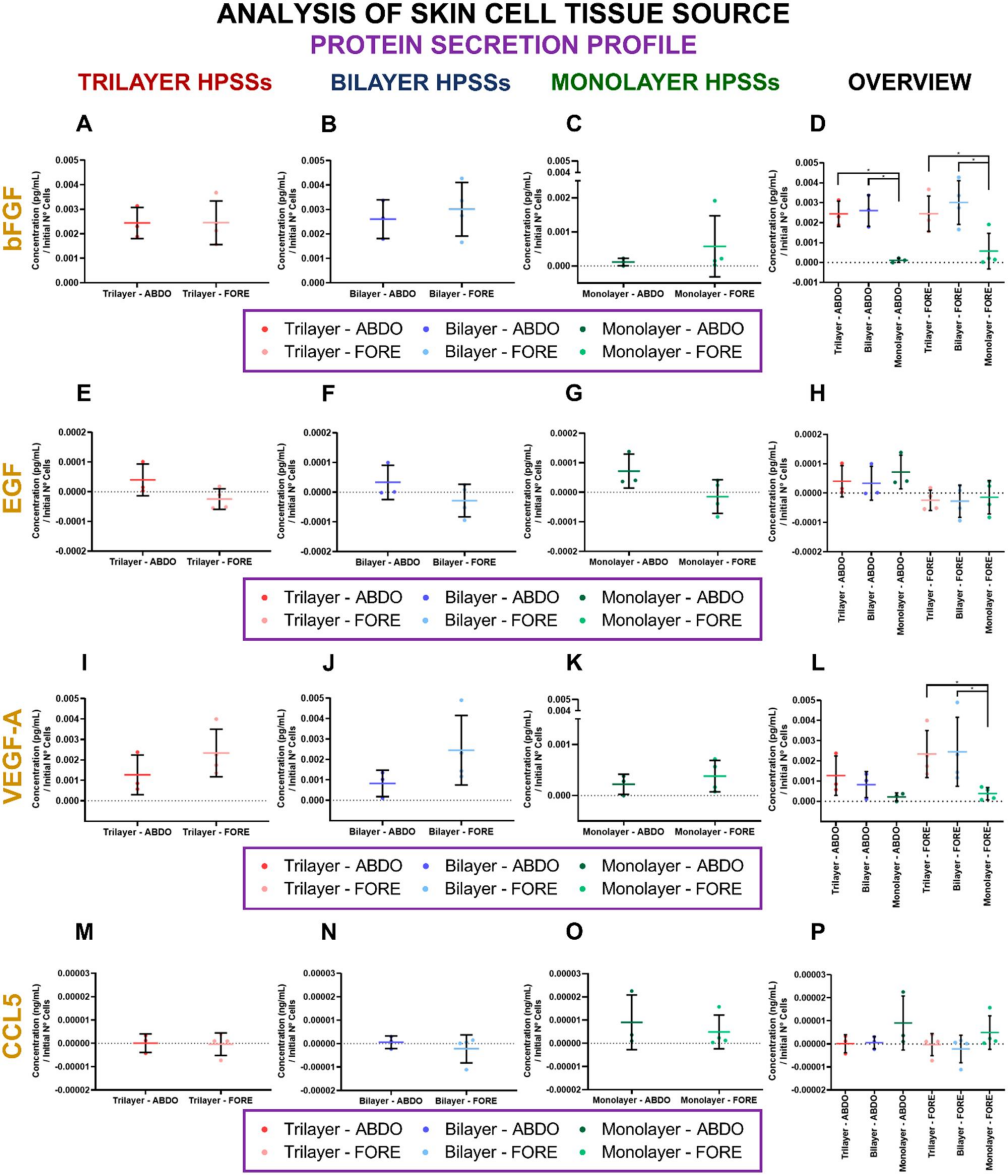
Protein secretion profile of human plasma-based skin substitutes (HPSSs) manufactured with several skin cell tissue sources. **bFGF secretion**: (**A**) Trilayer HPSSs; (**B**) Bilayer HPSSs; (**C**) Monolayer HPSSs; (**D**) Overall analysis of the different cellular HPSSs and their skin cell tissue source. **EGF secretion**: (**E**) Trilayer HPSSs; (**F**) Bilayer HPSSs; (**G**) Monolayer HPSSs; (**H**) Overall analysis of the different cellular HPSSs and their skin cell tissue source. **VEGF-A secretion**: (**I**) Trilayer HPSSs; (**J**) Bilayer HPSSs; (**K**) Monolayer HPSSs; (**L**) Overall analysis of the different cellular HPSSs and their skin cell tissue source. **CCL5 secretion**: (**M**) Trilayer HPSSs; (**N**) Bilayer HPSSs; (**O**) Monolayer HPSSs; (**P**) Overall analysis of the different cellular HPSSs and their skin cell tissue source. Statistical significance: *P values < 0.05, Welch’s *t*-test (if normal distribution) or Mann–Whitney test (if no normal distribution). Data are shown as mean value ± SD; *N* = 3 for abdominal skin source (*n* = 21) and *N* = 4 for foreskin source (*n* = 28). ABDO: abdominal skin source; FORE: foreskin source.

Regarding the protein secretion profile (bFGF, EGF, VEGF-A and CCL5) when direct comparison of each type of skin cell tissue source was analysed, no significant differences were reported for any of the cellular HPSSs studied (Figure 5). Curiously, the concentration of EGF and CCL5 secreted (after subtracting the control HPSS values) was close to zero or negative when foreskin cells were used (Figure 5E–G and M–O) which could mean that these cells require to capture or use them for several purposes, in a greater degree than abdominal skin cells.

Finally, the overview of the different cellular HPSSs for each of the skin cell tissue source analysed revealed that secretion of bFGF was significantly higher in trilayer and bilayer HPSSs compared with monolayer group for both cell sources (*P* values* < *0.05) (Figure 5D). In the case of VEGF-A the trend was the same, however only significant differences were reported when foreskin cells were applied (*P* values* < *0.05) (Figure 5L). For EGF and CCL5, no significant differences were observed between cellular groups neither abdominal nor foreskin cells were applied, but, interestingly, higher expression was reported in monolayer HPSSs (Figure 5H and P). In addition, histological appearance of the several cellular HPSSs manufactured with each type of skin cell tissue source revealed that no visual differences were reported (Supplementary Figure 2).

### Comparison of culture methodologies

Two culture methodologies used for manufacturing the cellular HPSSs were evaluated and compared. On the one hand, the submerged methodology, where keratinocyte medium fully covered the substitutes for 7–10 days to promote cell proliferation and growth. On the other hand, in the air/liquid interface approach, the substitutes were initially cultured under submerged conditions and then, the keratinocyte medium exempt of human epidermal growth factor was removed from the epidermal layer for a period of 10–13 days to induce terminal differentiation. Seven different skin cell populations were cultured under submerged conditions (SUB; *N* = 7) and five were evaluated by air/liquid interface methodology (ALI; *N* = 5). In the case of the protein secretion profile analysis, culture medium was collected when HPSSs were changed from SUB to ALI culture, so number of biological samples was 5 for both methodologies in this study. Moreover, an overview and analysis of the behavior of the different cellular HPSSs, depending on the culture methodology, was also provided in this section.

#### Cell viability and cell metabolic activity

**Figure 6.**
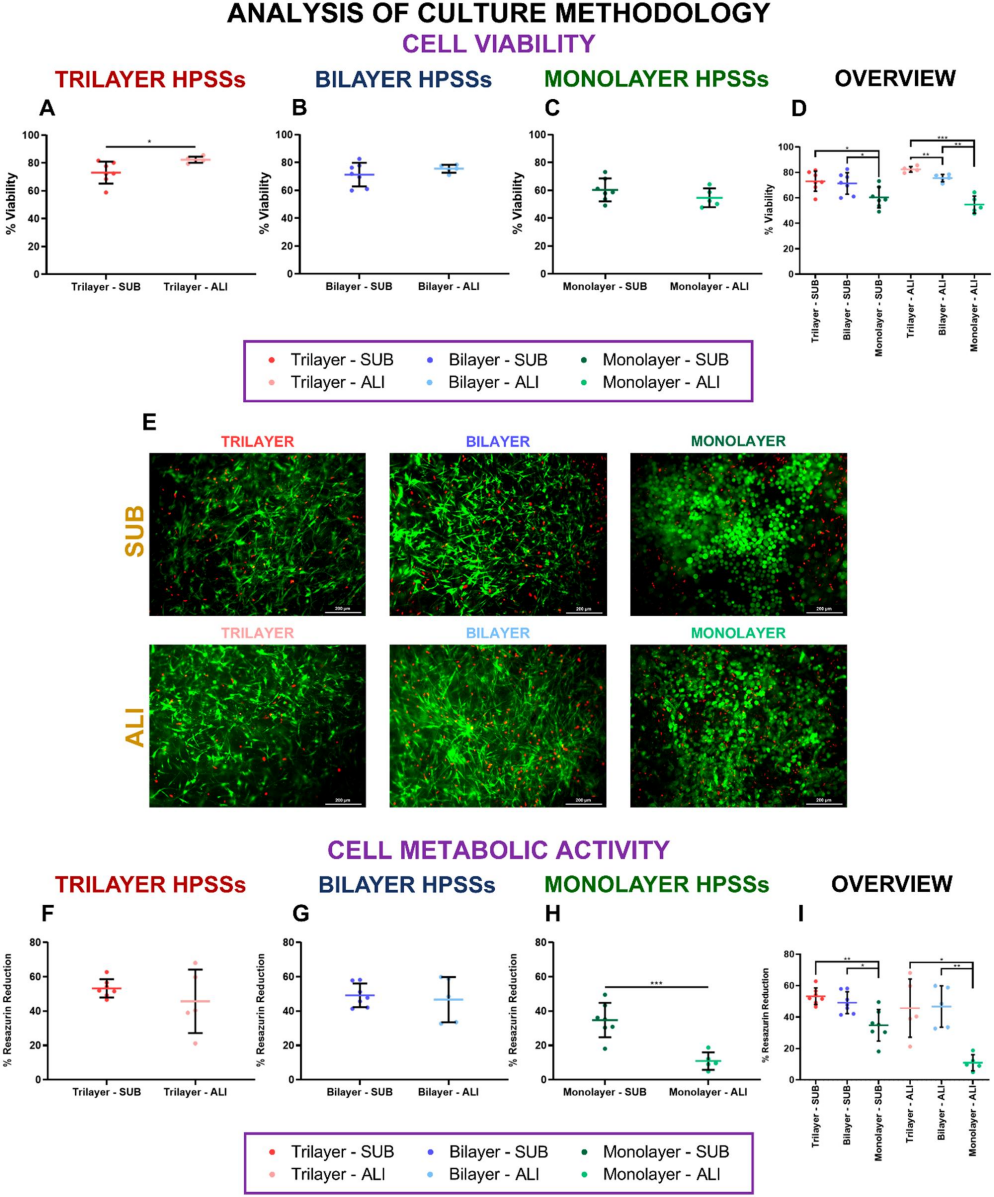
Cell viability and cell metabolic activity analysis of human plasma-based skin substitutes (HPSSs) cultured under submerged and air/liquid interface methodologies. (**A**) Cell viability of trilayer HPSSs; (**B**) Cell viability of bilayer; (**C**) Cell viability of monolayer HPSSs; (**D**) Overall analysis of the different cellular HPSSs and their culture methodology; (**E**) Representative microscope pictures of LIVE/DEAD™ viability/cytotoxicity assays; (**F**) Cell metabolic activity of trilayer HPSSs; (**G**) Cell metabolic activity of bilayer; (**H**) Cell metabolic activity of monolayer HPSSs; (**I**) Overall analysis of the different cellular HPSSs and their culture methodology. Scale bar: 200 µm. Statistical significance: *P values < 0.05, **P values < 0.01, ***P values < 0.001, Welch’s *t*-test. Data are shown as mean value ± SD; *N* = 7 for submerged condition (*n* = 49) and *N* = 5 for air/liquid interface condition (*n* = 35). SUB: submerged culture; ALI: air/liquid interface culture.

Cell viability was evaluated after dehydration process of the HPSSs culture under submerged and air/liquid interface (Figure 6). Comparison of both methodologies for each type of cellular HPSS revealed that cell viability of trilayer (Figure 6A) and bilayer (Figure 6B) was slightly higher when ALI culture was applied but decreased in monolayer HPSSs (Figure 6C). However, only a significant difference was observed in trilayer group (*P* values < 0.05) (Figure 6A).

Moreover, the comparison of the different cellular HPSSs for each culture methodology revealed that viability was higher in trilayer and bilayer than in monolayer HPSSs, although, this difference was higher in ALI culture (*P* values < 0.001 for trilayer group and *P* values < 0.01 for bilayer group vs. *P* values < 0.05 for both types of substitutes in SUB) (Figure 6D). In addition, a significant difference was also observed between trilayer and bilayer groups cultured and ALI conditions (*P* values < 0.01) (Figure 6D). Representative microscope pictures of LIVE/DEAD™ viability/cytotoxicity assays are included in Figure 6E. The morphological variability of the cells observed on the pictures, as in the previous cases, is due the nature of the cells used during the manufacturing process, and pictures selected to represent this analysis.

The cell metabolic activity analysis revealed that ALI culture decreased it in trilayer HPSSs (Figure 6F), bilayer HPSSs (Figure 6G) and monolayer HPSSs (Figure 6H). However, only significant differences were observed in monolayer HPSSs (from 34.8 ± 10.1% to 10.9 ± 5.1%; *P* values < 0.001) (Figure 6H). Analysis of the cellular HPSSs for each culture condition demonstrated similar values for trilayer and bilayer HPSSs in both, SUB and ALI methodologies (Figure 6I). However, when these values were compared with monolayer’s cell metabolic activity, significant differences were observed (*P* values < 0.05 and *P* values < 0.01) (Figure 6I).

#### Protein secretion profile

**Figure 7.**
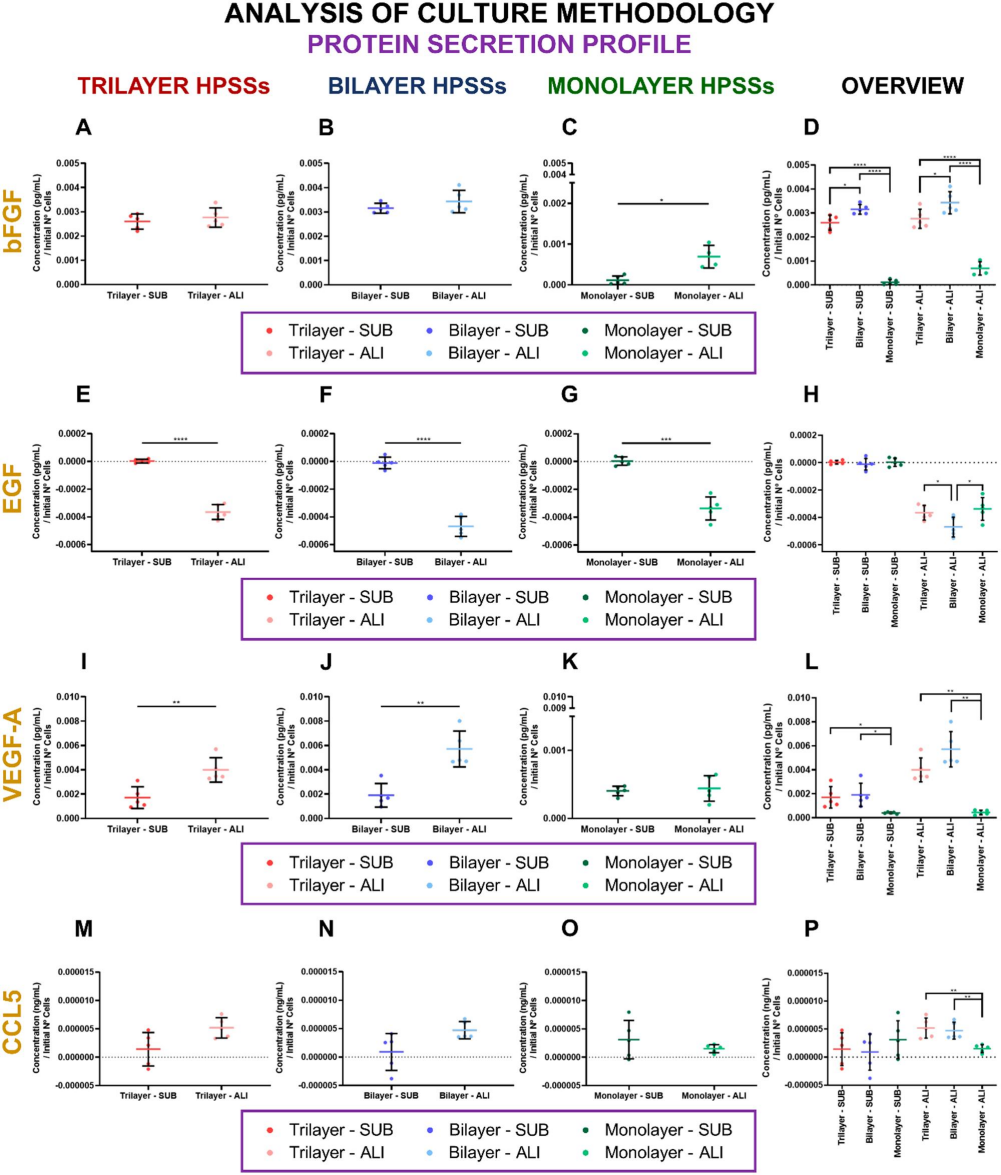
Protein secretion profile of human plasma-based skin substitutes (HPSSs) cultured under submerged and air/liquid interface methodologies. **bFGF secretion**: (**A**) Trilayer HPSSs; (**B**) Bilayer HPSSs; (**C**) Monolayer HPSSs; (**D**) Overall analysis of the different cellular HPSSs and culture methodology applied. **EGF secretion**: (**E**) Trilayer HPSSs; (**F**) Bilayer HPSSs; (**G**) Monolayer HPSSs; (**H**) Overall analysis of the different cellular HPSSs and culture methodology applied. **VEGF-A secretion**: (**I**) Trilayer HPSSs; (**J**) Bilayer HPSSs; (**K**) Monolayer HPSSs; (**L**) Overall analysis of the different cellular HPSSs and culture methodology applied. CCL5 secretion: (**M**) Trilayer HPSSs; (**N**) Bilayer HPSSs; (**O**) Monolayer HPSSs; (**P**) Overall analysis of the different cellular HPSSs and culture methodology applied. Statistical significance: *P values < 0.05, **P values < 0.01, ****P values < 0.0001, Welch’s *t*-test. Data are shown as mean value ± SD; *N* = 5 for submerged (*n* = 35) and air/liquid interface (*n* = 35) conditions. SUB: submerged culture; ALI: air/liquid interface culture.

The analysis of the protein secretion profile (bFGF, EGF, VEGF-A and CCL5) of both methodologies completes the analysis of the previous section where the different secondary biomaterials were individually studied for SUB and ALI cultures (Figure 2). In this case, the condition of the secondary biomaterial was ignored and small differences were reported (Figure 7).

The secretion of bFGF was higher when ALI methodology was applied in trilayer (Figure 7A), bilayer (Figure 7B) and monolayer HPSSs (Figure 7C). However, only significant differences were observed in monolayer substitutes (*P* values < 0.05) (Figure 7C). Moreover, comparison of the cellular HPSSs in each culture methodology revealed that trilayer and bilayer substitutes secreted significantly higher amount of bFGF than the monolayer groups (*P* values < 0.0001), but also that bilayer HPSSs also secreted more amount of this factor than trilayer substitutes (*P* values < 0.05) (Figure 7D).

In the case of EGF, values were close to zero for SUB condition and negative when ALI was applied for all groups (Figure 7E–G). Significant differences were observed in all cases although the lower was reported in the case of monolayer HPSSs (*P* values < 0.001) (Figure 7G). An overview of the different groups for each culture methodology revealed that the values of the SUB culture were similar regardless of the cellular composition (Figure 7H). On the other hand, in the case of ALI, negative values were reported in all groups although the lowest value was observed in bilayer group, reporting significant differences compared with both, trilayer and monolayer HPSSs (*P* values < 0.05) (Figure 7H).

Regarding VEGF-A analysis, as in the case of bFGF, the secretion was higher when ALI methodology was applied and significant differences were reported in trilayer (*P* values < 0.01) (Figure 7I) and bilayer (*P* values < 0.01) (Figure 7J) HPSSs. Monolayer substitutes under ALI conditions also secreted higher amount of VEGF-A but without a statistically significant difference (Figure 7K). Comparison of the cellular HPSSs, for each culture condition, revealed that trilayer and bilayer substitutes secreted similar amount of VEGF-A in both cases, and significantly higher than the secretion reported by monolayer groups (*P* values < 0.05 in SUB and *P* values < 0.01 in ALI) (Figure 7L).

Finally, secretion analysis of the cytokine CCL5 indicated the ALI culture slightly increased the secretion of this protein in trilayer (Figure 7M) and bilayer (Figure 7N) groups. However, in monolayer groups, this increase in secretion of CCL5 was reported in SUB but, again, without a significant difference (Figure 7O). The overview of the different cellular HPSSs for each culture methodology indicated that similar secretion was reported in the case of SUB groups (Figure 7P). However, under ALI condition a significantly higher secretion was observed in trilayer and bilayer HPSSs compared with monolayer substitutes (*P* values < 0.01) (Figure 7P).

#### Histological appearance and epidermal thickness

**Figure 8.**
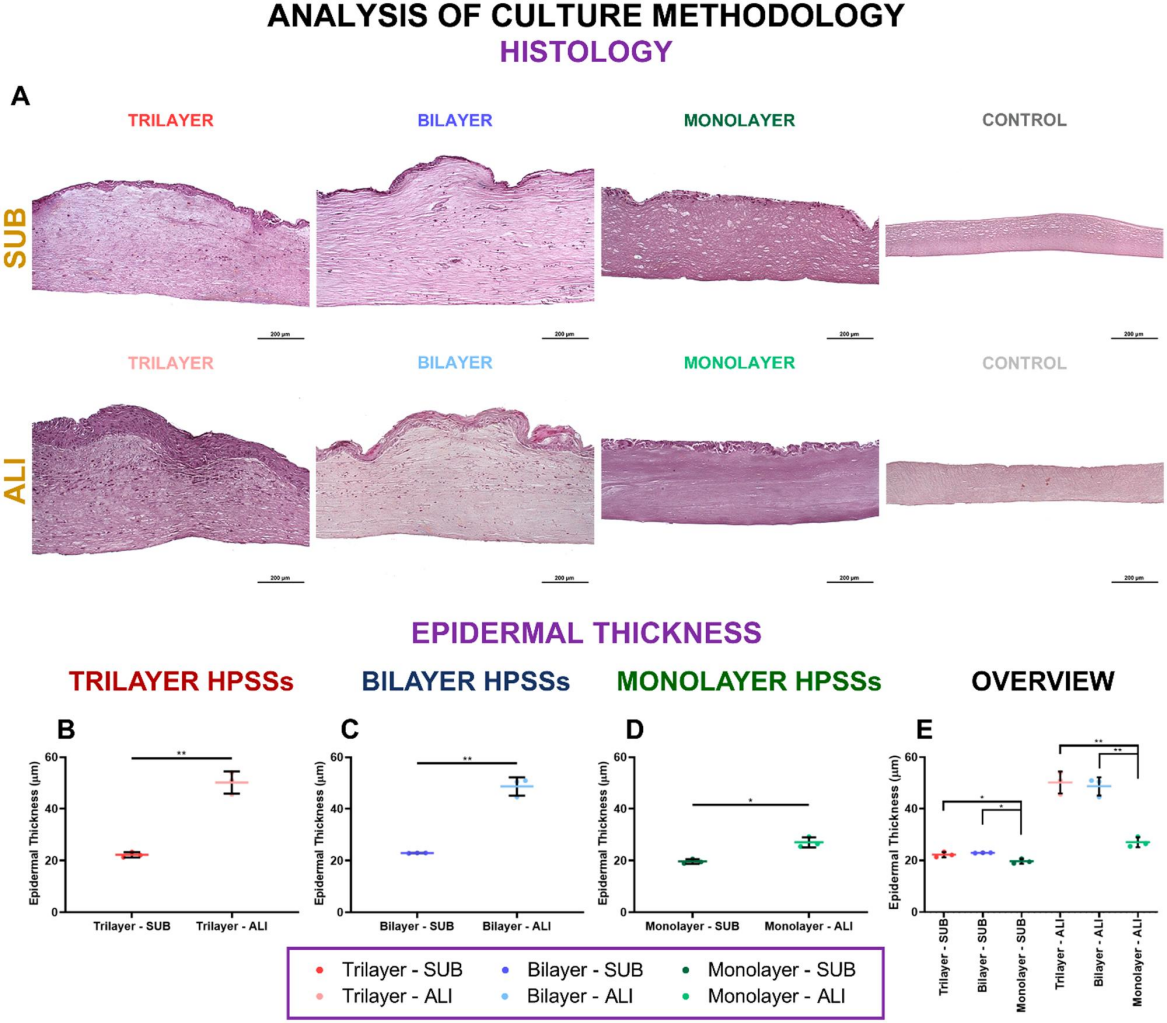
Histological appearance (hematoxylin/eosin staining) of human plasma-based skin substitutes (HPSSs) cultured under submerged or air/liquid interface methodologies and their epidermal thickness analysis. (**A**) Representative microscope pictures of hematoxylin/eosin staining; (**B**) Epidermal thickness of trilayer HPSSs; (**C**) Epidermal thickness of bilayer; (**D**) Epidermal thickness of monolayer HPSSs; (**E**) Overall analysis of the different cellular HPSSs and their culture methodology. Statistical significance: *P values < 0.05, **P values < 0.01, Welch’s *t*-test. Data are shown as mean value ± SD; *N* = 3 for submerged condition (*n* = 21) and *N* = 3 for air/liquid interface condition (*n* = 21). SUB: submerged culture; ALI: air/liquid interface culture. Fib/S: fibrin/serine; Fib/Fn: fibrin/fibronectin; Fib/Col: fibrin/collagen; Fib/Lam-1: fibrin/laminin-1; Fib/Lam-2: fibrin/laminin-2; Fib/HA: fibrin/hyaluronic acid; Fib: fibrin. Scale bar: 200 µm.

Histological appearance (Figure 8A) of the several cellular HPSSs manufactured under each type of culture methodology and secondary biomaterial revealed that a significantly thicker epidermis was observed when ALI methodology was applied in all groups [50.2 ± 4.3 µm vs. 22.2 ± 1.0 µm in trilayer HPSSs (*P* values < 0.01) (Figure 8B), 48.7 ± 3.5 µm vs. 22.9 ± 0.1 µm in Bilayer HPSSs (*P* values < 0.01) (Figure 8C) and 27.1 ± 1.9 µm vs. 19.7 ± 0.9 µm in monolayer HPSSs (*P* values < 0.05) (Figure 8D)]. Analysis and comparison of the epidermal thickness of the different cellular groups for each culture methodology applied revealed that in both cases, trilayer and bilayer groups presented a similar epidermal thickness, significantly thicker than the one from the monolayer substitutes (Figure 8E). However, this difference was higher when ALI strategy was applied (*P* values < 0.01) than when SUB methodology was evaluated (*P* values < 0.05) (Figure 8E).

## Discussion

The *in vitro* study of several variations of the human plasma-based skin substitute model manufactured at our laboratory, regarding the biomaterial and cellular composition and the manufacturing process, has provided useful information to increase the knowledge of this type of skin substitutes.

Firstly, the combination of human plasma with native biomaterials such as collagen [33, 57–59], hyaluronic acid [35, 60, 61], fibronectin [60] and laminin [62] had been previously studied for different purposes. However, no comparative *in vitro* or *in vivo* studies exist evaluating these different biomaterials’ compositions for HPSS manufacture. The results of this study demonstrated that no *in vitro* differences were observed regarding the secondary biomaterial added for any of the several conditions studied. Therefore, the majority component of the scaffolds, human plasma, determines their biological *in vitro* properties, as previously reported in a study where type I collagen was used as main component of bilayer TESSs and combined with several secondary biomaterials, demonstrating that relative cell viability was similar at different time points regardless of the addition or not of these biomaterials, even at different concentrations [63]. On the other hand, these results indicate that the role of the secondary biomaterials could be more interesting *in vivo* or depending on the dermatological pathology due to their wound healing properties [64] or their natural presence into native human skin. Interestingly, the combination of some of them to develop more complex dermal matrix has been already described to develop multicellular bioprinted substitutes that support skin regeneration *in vivo* [65].

Regarding the manufacturing process, the application of a partial dehydration process, also referred as plastic compression, has been described as a method to increase the mechanical properties of the hTESSs, for an improved clinical handling [47, 48]. The effect of this process in cell viability has been already evaluated in hydrogels composed of rat-tail type I collagen, demonstrating that its application resulted in a viability reduction of approximately a 10% [66]. These results are in line with the ones reported here, where the partial dehydration process reduced cell viability by ∼10%, ∼10% and ∼6% in trilayer, bilayer and monolayer groups, respectively. However, the viability of those substitutes with embedded fibroblasts and hAT-MSC was around 72%, which differs from the ∼80% previously reported for rat-tail I collagen [49]. This could be explained by the higher stiffness of collagen, demonstrated by comparing its compressive modulus value (1.5 ± 0.36 MPa) [66] with the one of human plasma/fibrin hydrogels (0.01 MPa) [46]. Therefore, collagen preserves cell viability much better when compression is applied and increase the concentration of this biomaterial in this HPSS model should be considered.

In terms of skin cell tissue source used for the HPSS manufacture, most of the previous studies have focused on the donor’s age and their influence in cell proliferation and epidermal thickness, indicating that neonatal or young donors may be of advantage due to their regenerative nature [67–70]. However, other studies indicated that the influence of the donor in general is higher in terms of cell proliferation than a specific age or anatomical location of the skin samples [63]. Those studies that directly studied the influence of the donor’s tissue source demonstrated that its influence is higher in keratinocyte proliferation and for *in vitro* culture [71], rather than for fibroblast culture [72] or hTESS manufacture, as corroborated in this research, where although small differences were observed when abdominal skin or foreskin cells were used for HPSS manufacture, none of them were significant.

Regarding the protein secretion profile of the different cellular human HPSSs manufactured, either abdominal skin or foreskin cells, some interesting results were reported. This analysis is determined by the use of the human plasma as a hydrogel, which contains several useful proteins that facilitate cellular activities and enhance deposition of a new ECM [1, 73]. However, the addition of the different cell types also determines the protein profile. The development of trilayer and bilayer substitutes, e.g. increased the release of bFGF and VEGF-A, which could be explained by the fact that bFGF is released by both fibroblasts (mainly) and keratinocytes [74, 75] and also, although in a minimum quantity, by hAT-MSCs [76], whereas, in the case of VEGF-A there is a cumulative secretion by keratinocytes [77], fibroblasts [78] and hAT-MSCs [76]. Interestingly, the use of human plasma/fibrin as scaffold has a paracrine effect that increases the secretion of VEGF compared to other scaffolds [79]. The release of these factors has clinical implications, since bFGF is known to stimulate collagen production and reduce fibrosis and scar formation [56], while VEGF-A initiates endothelial sprouting to promote angiogenesis [54].

In contrast, for EGF and CCL5, values were close to zero or negative which means that they were captured by the cells and, moreover, higher values of secretion were found in monolayer HPSSs. The explanation of this is that EGF is mainly captured by the fibroblasts for stimulating the production of collagen mainly in young populations [80]. While for CCL5, the higher secretion of this factor by monolayer than bilayer substitutes has been previously demonstrated [81], since stimulates *in vitro* the mobilization of fibroblasts [82] and hAT-MSCs [83]. In the particular case of the *in vivo* role of CCL5, although it is known to recruit macrophages to the wound during the inflammation phase [55], the lower release of this factor once the HPSSs are engrafted could be positive to avoid the chemoattraction of lymphocytes [84], which if it is imbalanced could lead to fibrosis and therefore, to an inadequate wound resolution [85].

Finally, the study of two culture methodology strategies for the manufacture of HPSS demonstrated that few significant differences in terms of cell viability and metabolic activity were observed. Regarding the cellular composition, like in all viability studies here described the value of monolayer HPSSs, in both SUB and ALI, was significantly lower compared with their corresponding trilayer and bilayer HPSSs. Moreover, these results were in line with the cell metabolic activity analysis, where lower values were reported for monolayer groups, especially when ALI was applied. This means that ALI affects monolayer substitutes or cultures in a higher degree, as previously observed in a study with the A549 epithelial cell line [86], and moreover, promotes early differentiation of epithelial cells instead of proliferation [87], corroborated by a significantly higher epidermal thickness in all groups. Furthermore, the cell differentiation is a process that maintains cell metabolic activity in contrast to when proliferation is promoted, where it is increased [88] as also observed. Interestingly, the increased values of epidermal thickness when ALI was applied, especially in trilayer HPSSs (50.2 ± 4.3 µm), were in line with the values previously described for human epidermis, which ranges from 50 to 100 µm in most of the anatomical regions of the body (except palms and soles) [89, 90]. Therefore, the application of ALI should be preferred when a better imitated human skin wants to be developed.

However, interesting results were reported with regard the protein secretion profile. In this context, the release patterns were similar in trilayer, bilayer and monolayer HPSSs for bFGF and VEGF-A growth factors, although higher when ALI was applied, due probably to the longer culture time. In the case of EGF, lower values (negative) were reported in all groups for ALI methodology, which could be due to the suppression of this factor from the culture medium to promote epithelial differentiation. This means that the factor which is present in the own plasma and produced by the own cells [91–93] is captured by the fibroblasts to produce collagen [80], whereas, in the case of submerged methodology, EGF is already available into the culture medium. Finally, the secretion of CCL5 in ALI methodology was the one that showed a different pattern of secretion compared to SUB strategy, regarding the cellular HPSS groups. In this last, a lower secretion was observed in trilayer and bilayer HPSSs compared with the monolayer group, the opposite behavior of the ALI condition. This could be explained because CCL5 is secreted and captured by all types of cells here used [94–97], so trilayer and bilayer groups under ALI strategy are able to release CCL5 but this is not captured by the keratinocytes, due to the lack of contact between them and the culture medium, thereby increasing the amount of this factor measured. Therefore, ALI increased the production and release of factors that enhance the wound healing process, such as bFGF and VEGF-A, but the manufacture is more time-consuming, something that could be important for the treatment of severely wounded patients with large-sized burns. In those cases, the engraftment of a less differentiated HPSS developed by SUB application has been demonstrated to be useful [19, 20] and should be consider depending on each specific case.

### Limitations

Overall, the lack of *in vitro* differences regarding the biomaterials used could indicate the necessity of evaluated them *in vivo*, to determine their specific function and role in wound healing. In the same way, the combination of different biomaterials and the increase of their concentration could be factors to consider developing more mimetic and dermis-like scaffolds. Regarding the variability of some of the results here showed, since primary human cells were directly isolated and cultured from several donors, and moreover, there are some processes, such as partial dehydration, that are manual procedures, this could explain the higher standard deviation observed in some cases. However, due to the large number of experiments and conditions analysed, this study is an interesting first approach towards the optimization of the HPSS models.

## Conclusions

On balance, the *in vitro* analysis of several subtypes of the clinical HPSS manufactured at our group revealed that the human plasma/fibrin determines their *in vitro* biological properties more than the secondary biomaterial included. Moreover, the use of skin cell populations from different tissue sources neither has a highlighted impact in the properties of this model. However, the manufacture of several cellular substitutes reported significant differences regarding the cell types included, indicating that better clinical outcomes are expected with trilayer and bilayer substitutes. In terms of culture methodology, the application of the air/liquid interface strategy generally increases the secretion of proteins that are useful in skin wound healing process and better histological structure is developed. Nevertheless, depending on the time available for its manufacture and the urgency, the submerged methodology is also a useful strategy due to the shorter production time required. Finally, the effect of the partial dehydration process applied after culturing process decreases cell viability of the HPSSs, but this reduction is minimal and clinical handling is improved.

Therefore, the results of this *in vitro* study reveal that this HPSS model is robust and reliable and that the several subtypes here analysed could be promising clinical approaches depending on the dermatological disease, although *in vivo* studies should corroborate this statement.

## Supplementary Material

rbae115_Supplementary_Data

## Data Availability

The authors declare that the data supporting the findings of this study are available within the article and its supplementary information files.
