# Supplementary material for: Biological properties and characterization of several variations of a clinical human plasma-based skin substitute model and its manufacturing process
Source: Regen Biomater. 2024 Sep 26;11:rbae115. doi: 10.1093/rb/rbae115 (PMC11513639; doi:10.1093/rb/rbae115)
Supplement: rbae115_Supplementary_Data [file rbae115_supplementary_data.zip › Supplementary_Tables.docx]

***Supplementary Table 1****. Information about secondary biomaterials used for the human plasma-based skin substitutes (HPSSs) manufacture.*

| Biomaterial | Company | Final Concentration | Abbreviation |
| --- | --- | --- | --- |
| Serine  (S) | Biogelx^TM^, Lanarkshire, UK | 670 μg/mL | S |
| Fibronectin  (RGD) | Biogelx^TM^, Lanarkshire, UK | 740 μg/mL | Fn |
| Collagen  (GFOGER) | Biogelx^TM^, Lanarkshire, UK | 780 μg/mL | Col |
| Laminin  (IKVAV) | Biogelx^TM^, Lanarkshire, UK | 790 μg/mL | Lam-1 |
| Laminin  (YIGSR) | Biogelx^TM^, Lanarkshire, UK | 910 μg/mL | Lam-2 |
| Hyaluronic Acid | Fidia Farmaceutici S.p.A., Abano Terme, Italy | 750 μg/mL | HA |
| Information between brackets indicates the combination of amino acids used for biomaterial production and that resembles the physiological motif structure of the different peptides described.  Amino acid abbreviations: A: Alanine, D: Aspartic acid, E: Glutamic acid, F: Phenylalanine, G: Glycine, I: Isoleucine, K: Lysine, O: Hydroxyproline,  R: Arginine, S: Serine, V: Valine, Y: Tyrosine. | | | |

***Supplementary Table 2****. Reagents and volume for Trilayer human plasma-based skin substitutes (HPSSs) manufacture.*

|  | Reagents for hypodermal layer | Volume | Reagents for dermal layer | Volume |
| --- | --- | --- | --- | --- |
| Solution 1 | Calcium Chloride  100 mg/mL (CaCl_2_)  (BBraun, Melsungen, Germany) | 0.060 mL | Calcium Chloride  100 mg/mL (CaCl_2_)  (BBraun, Melsungen, Germany) | 0.060 mL |
|  | Water for injection  (Fresenius Kabi, Bad Homburg, Germany) | 0.0335 mL | Water for injection  (Fresenius Kabi, Bad Homburg, Germany) | 0.0335 mL |
| Solution 2 | Human Plasma | 2.075 mL | Human Plasma | 2.075 mL |
|  | MSC medium + hAT-MSCs | 0.156 mL | Fibroblast medium + human fibroblasts | 0.156 mL |
|  | Secondary Biomaterial* | 0.125 mL | Secondary Biomaterial* | 0.125 mL |
|  | Tranexamic Acid  100 mg/mL  (MEDA Pharma, Bad Homburg, Germany) | 0.0505 mL | Tranexamic Acid  100 mg/mL  (MEDA Pharma, Bad Homburg, Germany) | 0.0505 mL |
| Hypodermal layer was manufactured 3-4 hours before dermal layer to allow a partial hydrogel formation to clearly differentiated both layers but allowing their integration.  *In the case of the HPSSs manufactured without any secondary biomaterial, the same extra volume of human plasma was added. | | | | |

***Supplementary Table 3****. Reagents and volume for Bilayer, Monolayer and Control human plasma-based skin substitutes (HPSSs) manufacture.*

|  | Reagent for dermal layer | Volume |
| --- | --- | --- |
| Solution 1 | Calcium Chloride  100 mg/mL (CaCl_2_)  (BBraun, Melsungen, Germany) | 0.120 mL |
|  | Water for injection  (Fresenius Kabi, Bad Homburg, Germany) | 0.067 mL |
| Solution 2 | Human Plasma | 4.150 mL |
|  | Fibroblast medium + human fibroblasts (Bilayer TESS)  or no cells (Monolayer and Control HPSSs) | 0.312 mL |
|  | Secondary Biomaterial* | 0.250 mL |
|  | Tranexamic Acid  100 mg/mL  (MEDA Pharma, Bad Homburg, Germany) | 0.101 mL |
| For Bilayer HPSSs addition of human fibroblasts was required for dermal layer, however for Monolayer and Control HPSSs only fibroblast medium was required.  *In the case of the HPSSs manufactured without any secondary biomaterial, the same extra volume of human plasma was added. | | |
