# Supplementary figures and images for: Biological properties and characterization of several variations of a clinical human plasma-based skin substitute model and its manufacturing process
Source: Regen Biomater. 2024 Sep 26;11:rbae115. doi: 10.1093/rb/rbae115 (PMC11513639; doi:10.1093/rb/rbae115)

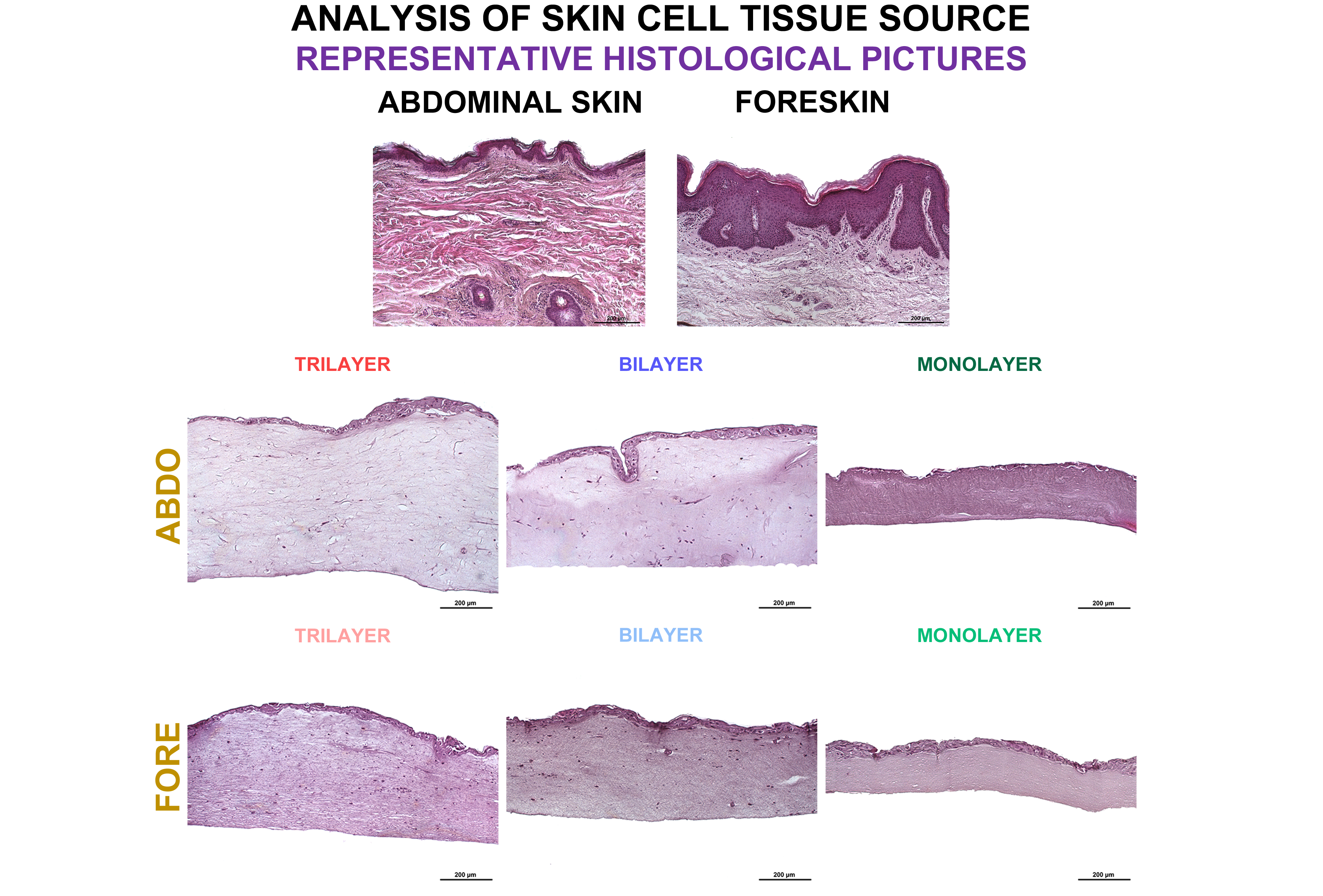

Supplement: rbae115_Supplementary_Data [file rbae115_supplementary_data.zip › Supplementary_Figure_2.tif]

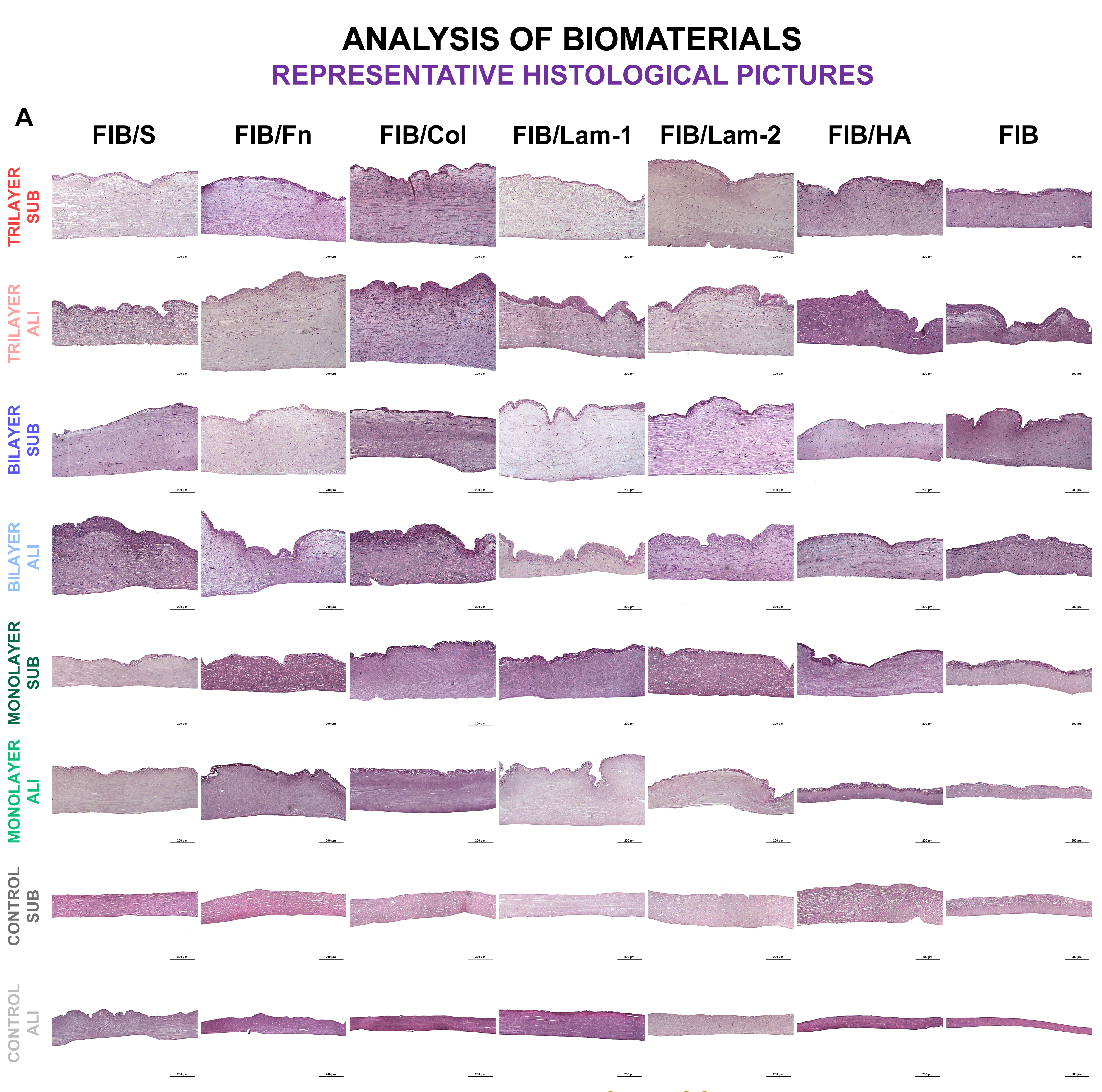

Supplement: rbae115_Supplementary_Data [file rbae115_supplementary_data.zip › Supplementary_Figure_1.tif]
